# COVID-19, social media, algorithms and the rise of indigenous movements in Southern Africa: perspectives from activists, audiences and policymakers

**DOI:** 10.3389/fsoc.2024.1433998

**Published:** 2024-10-16

**Authors:** Metolo Foyet, Brian Child

**Affiliations:** Department of Geography, University of Florida, Gainesville, FL, United States

**Keywords:** social media, indigenous activism, digital empowerment, algorithms, social movements, COVID-19, community leaders network, Southern Africa

## Abstract

**Introduction:**

The COVID-19 pandemic, as a holistic event of cultural trauma, significantly influenced social structures and behaviors globally. Under its impact, social movements leveraged digital platforms to sustain and amplify their causes, creating new forms of solidarity and resistance, and fostering a rise in digital and hybrid collective actions. Concurrently, social media thrived as a transformative tool for social change, revolutionizing communication, mobilization, and advocacy. Platforms like WhatsApp and X redefined traditional activism by enabling rapid information dissemination and facilitating global grassroots movements. This technological evolution has provided marginalized communities, including the indigenous peoples of Southern Africa, with a powerful voice. These communities face challenges such as land rights disputes, environmental degradation, and socioeconomic marginalization. Social media allows them to raise awareness, galvanize support, and engage with a broader audience beyond their geographical confines. The paper hypothesizes that social media plays a multifaceted role in supporting indigenous movements, by not only providing a platform for activists to organize and advocate, but also enabling engagement with the general public and influencing the perspectives and actions of policymakers and other audiences. Through the lenses of rural or indigenous activists who leverage these digital platforms to drive change, audiences who consume and interact with digital content and feeds, and policymakers who are increasingly mindful of the power of social media narratives, this paper aims to understand the complex interplay of forces that shape the trajectory of digital indigenism (indigenous digital activism).

**Methods:**

The paper employs a mixed-methods approach to investigate the influence of social media on social movements among indigenous communities in Southern Africa. The methodology incorporates (a) netnography and in-depth interviews to explore the experiences and strategies of indigenous activists, (b) the counterpublics framework to understand the formation and dynamics of indigenous digital activism, and (c) the Technology-Media-Movements Complex (TMMC) as a theoretical anchor to analyze the interplay between technology, media, and social movements. The case study of the Community Leaders Network (CLN) of Southern Africa is used to contextualize the findings.

**Results:**

Findings reveal that indigenous activists recognize the power of social media in amplifying their voices but use these platforms out of necessity rather than preference. They find social media solutions often misaligned with their contextual needs, citing concerns over platform constraints, privacy issues, cultural insensitivity, superficial engagement metrics, and breaches of consent. Additionally, they reckon that the global emphasis on social media engagement can divert focus from essential field activities that directly benefit local communities, causing social media fatigue. It was also revealed that trying to convey practical information to an audience with preconceived notions is incredibly time-consuming and often feels like an endless loop for indigenous activists. Subsequently, they expressed a desire for platforms that consider users’ mental well-being in their architectural design and incorporate cultural and linguistic practices, suggesting a preference for digital environments that are more aligned with values and modes of communication that contrast with western models.

**Discussion:**

The results underscore social media’s complex role in indigenous movements, highlighting its empowering potential while also presenting significant challenges due to algorithms and platform dynamics. While the ability to share stories, disseminate information about rights abuses, and mobilize support has significantly transformed social movement dynamics in rural communities, social media’s potential for advocacy and mobilization is tempered by challenges that can limit their effectiveness. The findings highlight a pressing need for social media innovations that resonate with indigenous cultural identities, ensuring that their narratives are disseminated in a manner that faithfully preserves their authenticity. The paper discusses the implications of these findings for policymakers, activists, audiences and technology developers, emphasizing the importance of creating digital spaces that are culturally sensitive and supportive of indigenous activism.

## 1 Introduction

The COVID-19 pandemic, as a holistic event of cultural trauma, has significantly influenced social structures and behaviors globally, reshaping how communities interact and mobilize. It highlighted the importance of digital platforms as essential tools for maintaining social cohesion and advancing activism during times of physical distancing and restricted movement. Under its impact, social movements leveraged digital platforms to sustain and amplify their causes, creating new forms of solidarity and resistance, and fostering a rise in digital and hybrid collective actions.

Concurrently, social media stands as a formidable platform, with over 5.17 billion users worldwide (Sprout Social, 2024), offering unparalleled opportunities for raising awareness and mobilizing support at a scale never seen before. Yet, harnessing this digital momentum to instigate concrete policy changes in conservation and achieve substantial real-world impacts on rural livelihoods remains a significant hurdle. The primary challenge is effectively bridging the digital activism prevalent on social media with the tangible, on-the-ground efforts of community conservation and grassroots movements. This is especially crucial for indigenous communities, who, despite representing less than 6% of the global population, are guardians of 80% of the world’s biodiversity and account for about 19% of the extreme poor (World Bank, 2023). These communities are pivotal in conservation efforts but often find their voices sidelined in the broader environmental discourse (Louis, 2024), and critically under-represented specifically in conservation-based online discourse, highlighting an urgent need to ensure digital empowerment translates into genuine advocacy and support for their rights and traditional ecological knowledge (TEK). Addressing this need, the Community Leaders Network (CLN) of Southern Africa, a Namibia-based burgeoning conservation movement led by indigenous representatives from 15 Southern African nations, is committed to transforming this narrative. Emerging from the shortcomings of exclusionary conservation models and inspired by the achievements of Southern African-thought community- based natural resource management (CBNRM) model, CLN’s mission is to elevate the influence of local communities in conservation: “We are the custodians of our natural resources and the key to its sustainability. Our voices matter” is boldy inscribed on their website’s homepage (CLN, n.d.). Their strategy focuses on amplifying the voices of local communities through enhanced community participation and influencing policy negotiation, development, and implementation at national, regional, and international levels. CLN is dedicated to ensuring that decision-making processes are well-informed and reflective of the local communities’ needs and rights, enabling them to manage and derive benefits from their natural resources effectively (CLN, n.d.).

While there is a wealth of studies on social movements at large (Piven and Cloward, 1978; Melucci, 1980; Habermas, 1981; Cohen, 1983; Smith, 1987; Castells, 1989; McAdam et al., 1997; Ganz, 2001; Della Porta and Diani, 2006; Cox and Nilsen, 2007; Pleyers, 2024), the existing body of research barely covers what we coin “digital indigenism” (indigenous digital activism) and the role, limitations, successes and failures of digital technology from the perspectives of those involved in, affected by, influencing, and at the forefront of this type of activism. For instance, studies might focus on the impact of social media on mobilizing support or creating awareness on a global scale, but the unique ways in which rural communities in Southern Africa harness these digital tools for their social, political, or environmental movements are scarcely documented or understood.

Although there is substantial research on grassroots movements (Moyo and Yeros, 2005) in Latin America—including Brazil’s Landless Workers’ Movement (MST) (Wolford, 2004) and Guarani- Kaiowá Resistance (Urt, 2016; Mondardo, 2022; Ioris, 2022), Mexico’s Zapatista Movement (Ronfeldt et al., 1999; Russell, 2005; Inclán, 2018), Chile’s Mapuche Struggles (Haughney, 2007; Meza, 2009; Carruthers and Rodriguez, 2009; Haughney, 2012), Colombia’s Indigenous Peoples’ Movements (Ulloa, 2013), Peru, Bolivia and Venezuela’s Andean Indigenous Movements (Van Cott, 2003; Korovkin, 2006), Guatemala’s Maya Movement (Fischer and Brown, 1996; Arias, 2006; Warren, 2018), The Amazonian Indigenous Rights Movement (O’Connor, 1994; Aviles, 2012; Bolaños, 2014; Martin, 2014); Europe—including Spain’s Doñana’s environmentalist protests (Rodríguez-Giralt, 2011) and Scandinavia’s Sámi Rights Movement (indigenous to the Arctic areas of Norway, Sweden, Finland, and Russia) (Lantto and Mörkenstam, 2008; Allard, 2011; Bunikowski, 2013; Greaves, 2018); and Asia—including Nepal’s Land Rights Movement from Below (Karki, 2002), the African context has not received as much scholarly attention. Furthermore, the specific exploration of the culture factor in online activism is markedly underrepresented in academic literature. This oversight is notable, especially considering the increasing relevance of digital platforms in global activism, and the transformative potential of digital technologies in amplifying marginalized voices and facilitating grassroots movements; specifically when local people are so greatly affected by decisions—made in global fora like CITES and IUCN—that substantially affect their livelihoods and the wildlife they live with.

Reductive and simplistic approaches toward cognition, intelligence, human behavior and social systems remain the default, especially in Western sciences (Birhane, 2021). Implicit assumptions underlying conservation science, traditional media-movement inquiry and contemporary media discourse are no different. Complex, messy, contextual, dynamic, multivalent, and ambiguous phenomena are often reduced down to their abstract “essence” or single representation (Birhane, 2021). Part of the problem with systems that sort, classify, and predict human behavior and social phenomena, springs from this simplistic and limited understanding. The other part of the problem lies in the fact that voices from studied communities and of those impacted by the topics examined are nearly absent in scholarly and decision-making circles. To that end, this paper leans on (1) introducing disregarded actors in the literature, and (2) adding insights from (their) non-dichotomic approaches to characterizing cognition, human behavior, and social systems with the aim of revealing both the scientific and ethical limitations of societal and machine classification, perception and prediction.

### 1.1 Methodology

#### 1.1.1 Netnography

Developed by Robert Kozinets in the 1990s, netnography is a term derived from “internet” and “ethnography.” The qualitative research method adapts the traditional ethnographic study of cultures and communities to the context of the online world (Kozinets, 1998). It involves observing and analyzing the behavior and interactions of individuals within online communities, forums, social media platforms, and other digital spaces (Bowler, 2010). This method is particularly valuable for understanding the social dynamics, cultural norms, and discourses that unfold in virtual environments, which are increasingly integral to people’s daily lives (Bartl et al., 2016; Fenton and Procter, 2019).

The application of netnography in the context of this study aligns closely with Gilbert Ryle’s concept of “thick description” (Gilbert, 2001; Kozinets and Gambetti, 2021). This approach goes beyond simply reporting social media content, instead offering a deep, contextual understanding of the social actions and meanings behind the content from the perspectives of the activists, in this case, community leaders from Southern African indigenous communities.

If we take Lugosi and Quinton’s (2018) idea of “more-than-human netnography” seriously, netnography also includes other acts and actors beyond posts and those who post them, expanding to explain, for instance, the meaning of a platform or a site’s buttons or forms, to interpret the hidden exclusions of algorithms or decipher the chattering of bots (see Chapter 16). Where the algorithm goes, the astute netnographer will follow, chasing down what it allows, what it removes, what it randomizes, whose interests it exalts, whose it exploits, and whose are excluded altogether. – Kozinets and Gambetti, 2021

For example, the study discusses how the CLN used platforms like WhatsApp to maintain communication and mobilize support during the COVID-19 pandemic. This usage of social media during the COVID-19 pandemic can be contextualized within the broader challenges they faced, such as limited physical mobility and increased digital reliance. The study equally discusses how the activists’ online activities were influenced by offline events, community interactions, and personal experiences, offering a comprehensive view of their activism. It also details the practical and emotional challenges faced by these activists (Demertzis, 2021), providing a nuanced understanding of their digital interactions.

#### 1.1.2 Online interviews

Another contribution has been to engage in inaugural interviews to investigate the new conservation movement that is CLN as a case study for academic inquiry. To that end, four-hour long comprehensive interviews were conducted, and conversations spanning varied lengths were held with CLN founders, leaders, members, friends and community beneficiaries to gain insights into CLN’S apparatus and the experiences and perceptions of stakeholders involved. The interviews were analyzed using thematic and discourse analyses. The first analysis was used to identify, analyze, and report patterns within the data collected from the interviews. These patterns informed the themes under which the Results and Discussion sections were organized. The second analysis was used to examine the language, power relations, and underlying ideologies expressed (Creswell and Poth, 2018) during the interviews, helping to understand how activists articulate their experiences and the social and political contexts influencing their narratives. Thick description emphasizes interpreting social actions from the perspective of the actors themselves. Interviews with indigenous digital activists captured their personal insights, feelings, reflections, challenges and aspirations, offering a window into their worldview and enabling the study to grasp the symbolic meanings and cultural significance of digital activism for these individuals. This emotional and cultural depth enriched the thick description by providing a fuller picture of the activists’ motivations and the impact of their actions.

#### 1.1.3 Complementarity and integration of methods

Netnography captures the everyday interactions and public expressions within digital platforms, providing a broad understanding of how communities engage online. While this method reveals patterns, behaviors, and discourses that might be missed in interviews, the latter offers deeper, personal insights into the motivations, feelings, and lived experiences of individuals. They help explain the reasons behind observed online behaviors and provide context that netnography alone cannot uncover. Additionally, the outcomes of the observation and the interviews will be combined via (a) cross-validation: findings from netnography will be cross-validated with data from interviews. For example, observed trends in online activism will be discussed with interview participants to understand their underlying motivations and confirm the authenticity of online expressions; and (b) contextualization: interviews will provide context to the data collected through netnography. For instance, if netnography reveals a particular discourse trend on social media, interviews can help contextualize why this trend emerged and how it is perceived by the community.

### 1.2 General concept

#### 1.2.1 The counterpublics

The concept of counterpublics evolved as Nancy Fraser’s response to Jürgen Habermas’s notion of public sphere, which he described as a domain of social life where individuals come together to freely discuss and identify societal problems, and through that discussion influence political action (Habermas, 1991). Fraser, among other scholars, critiqued Habermas’ public sphere for being overly centered on a bourgeois, male-dominated discourse; thereby introducing the idea of “counterpublics” or alternative publics where marginalized groups create their own public spheres to discuss issues relevant to them (Fraser, 2014). In this paper, it has been adapted to analyze how social media creates both a digital public sphere (where the bourgeoisie dominates the narrative) and a digital counter public (where marginalized voices, particularly those of rural communities, can participate—although on an unequal footing—in broader societal dialogs, advocate for change, and influence policy).

### 1.3 Theoretical framework

#### 1.3.1 The technology-media-movements complex

**Figure 1:**
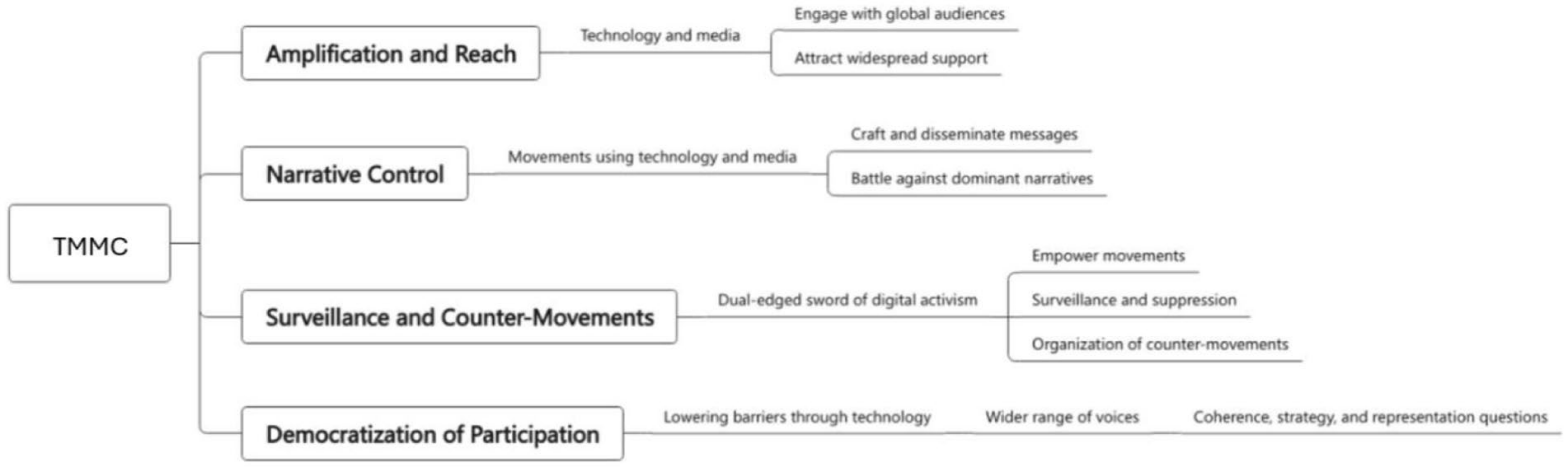
Implications of TMMC (Source, Author).

**Figure 2:**
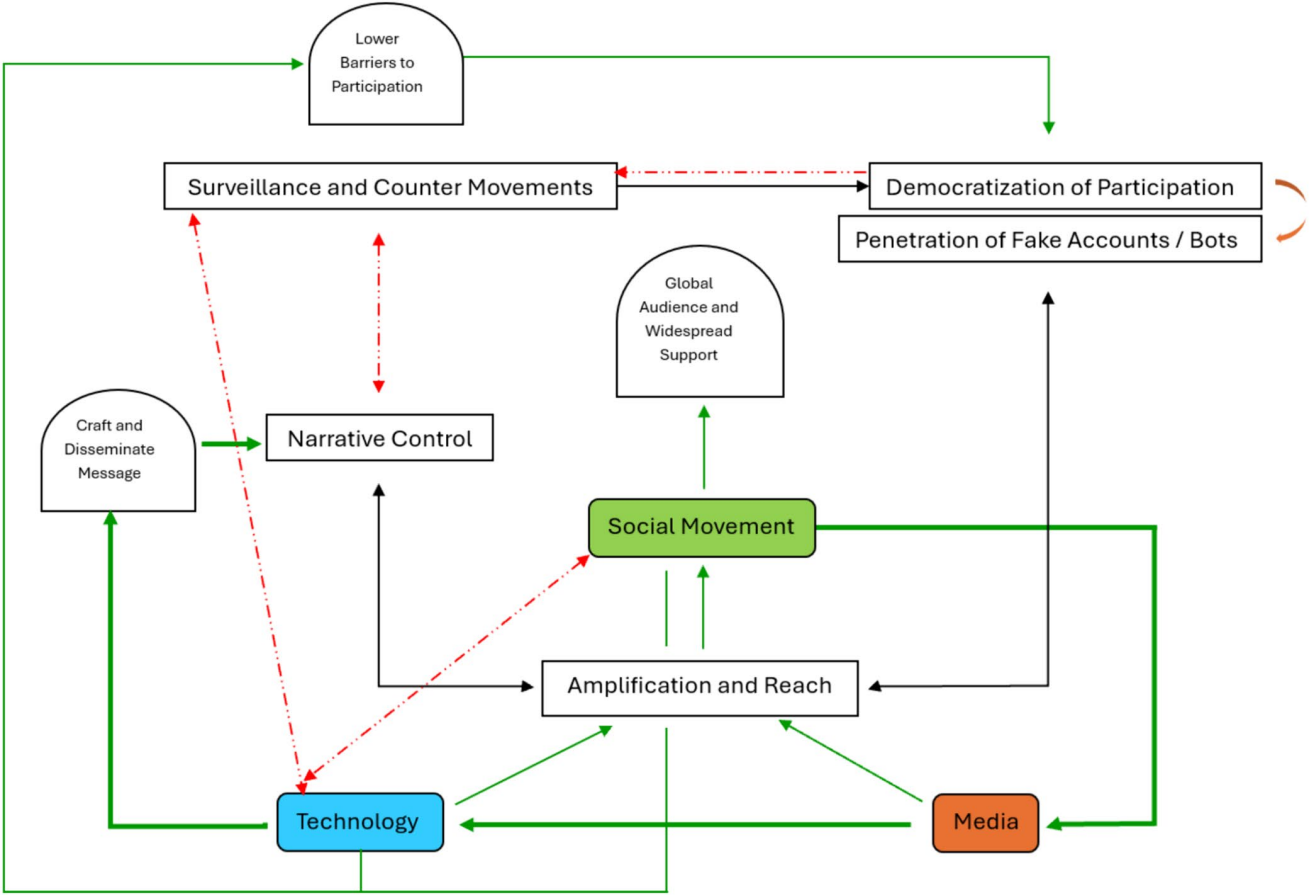
Intersections of TMMC (Source, Author).

TMMC provides a framework for understanding the modern landscape of activism, where technological tools, media dynamics, and movement strategies are deeply intertwined. It invites a critical examination of how these elements interact, the power dynamics at play, and the potential for social change in the digital age. This framework recognizes that technological advancements, media strategies, and social movements are not isolated phenomena but are deeply interconnected, each playing a pivotal role in the dynamics of activism and public discourse (Figures 1, 2; Table 1).

**Table 1:** Key components of the technology-media-movements complex (TMMC).

Category	Description
Technology	Provides the tools and platforms enabling new forms of communication, organization, and mobilization. From social *media* networks to messaging apps and beyond, technology lowers barriers to entry for participation in social *movements*, allowing for the rapid spread of information and the ability to coordinate action across geographies. It also offers innovative ways to bypass traditional media gatekeepers, democratizing the production and dissemination of content
Media	Encompasses both traditional outlets and new digital platforms, acts as the conduit through which information about social *movements* reaches broader audiences. It plays a critical role in framing issues, shaping public perceptions, and influencing the narrative around social movements. The relationship between media and movements is symbiotic yet complex, with *media* strategies being employed by activists to garner attention, while media outlets themselves seek to capture compelling stories that resonate with their audience
Movements	Represent the collective efforts of individuals and groups to advocate for change, resist injustice, or promote a cause. Movements *leverage both technology and media* to amplify their message, mobilize supporters, and engage in dialog (or confrontation) with power structures. The evolution of movements is significantly influenced by technological innovations and media dynamics, with strategies and tactics constantly adapting to the changing landscape

The diagram outlines the relationship between different components of social movements and the role technology and media play in this context.

- **Amplification and Reach** is at the base of the diagram (Figure 2), connected to both Technology and Media. It signifies that technology and media are essential tools for amplifying the message of a social movement, reaching a global audience and attracting widespread support.
- **Narrative Control**: Positioned above “Amplification and Reach,” it highlights the importance of crafting and disseminating a message effectively. Narrative Control is crucial for a social movement to maintain its objectives and perspectives in the public eye. Also, movements use technology and media to craft and disseminate their messages, often in opposition to dominant narratives (e.g., traditional media) or opposed by certain technological gatekeepers.
- **Surveillance and Counter-Movements**: This is to the left of “Narrative Control” and has a bidirectional arrow pointing between them. It suggests that while social movements seek to control their narrative, there is also a dynamic of surveillance and potentially the emergence of counter-movements that can challenge or suppress the original movement.
- **Democratization of Participation**: To the right of “Narrative Control,” it shows that as barriers to participation are lowered (through technology), more people can join and influence the social movement. This democratization can lead to a diversity of voices within the movement. Though this also raises questions about coherence, strategy, and representation, hence the orange curved arrow.
- **Penetration of Fake Accounts and Bots** exacerbates these issues by distorting genuine participation and discourse. These inauthentic actors might amplify misleading narratives, disrupt strategic messaging, and undermine the movement’s representation by creating noise and diluting the voices of genuine participants. This highlights the challenge of maintaining narrative integrity and effective communication in an increasingly democratized and digital landscape. As indicated by Poulakidakos, in the light of the discussion surrounding conspiracy theories and fake news, this drawback has to be addressed (Poulakidakos, 2024).

The green arrows indicate positive influence or support. Those pointing from “Technology” and “Media” to “Amplification and Reach” indicate that both facilitate the broad spread of the movement’s message. The red dashed arrows represent a negative or opposing relationship. That from “Democratization of Participation” to “Surveillance and Counter Movements” suggests that increased participation can also lead to greater scrutiny and the potential for opposing movements. That pointing from “Surveillance and Counter Movements” to “Narrative Control” signifies that surveillance and counter-movements can interfere with or attempt to take over the narrative controlled by the social movement and vice versa. Finally, that pointing from “Social Movement” to “Technology” to “Surveillance and Counter Movements” signifies that the same technologies that empower movements can also be used for surveillance, suppression, or the organization of counter-movements, introducing a dual-edged sword of digital activism. In the center, the “Social Movement” box is where all these elements converge, indicating that it is at the heart of these interactions. The movements use technology and media to amplify their reach and control their narrative while navigating the challenges of surveillance and counter-movements, all within the broader context of increased participation from the public.

## 2 Results

Indigenous communities in Southern Africa have a long history of resistance, stemming from colonial legacies, battles over land rights, and the struggle for cultural preservation and self- determination. These movements—grounded in daily interactions and use of wildlife and domestic animals, where the power over wildlife is controlled, urbanized, romanticized and disconnected (trade of wildlife hunting, green militarization)—have evolved from localized protests and community gatherings to more organized national and transnational campaigns, thanks to the advent of global connectivity and the spread of digital platforms. This historical trajectory of activism is marked by a transition from traditional forms of communication to leveraging the digital landscape’s potential to amplify voices and mobilize support. Additionally, the COVID-19 pandemic, as a holistic event of cultural trauma,^1^ has significantly influenced social structures and behaviors globally. According to Demertzis and Eyerman, it has reshaped the way communities interact, fostering a rise in digital and hybrid social movements (Demertzis and Eyerman, 2020). These movements, both online and offline, have adapted to the constraints imposed by the pandemic, creating new forms of solidarity and resistance. For instance, emerging literature explores how micro social movements have leveraged digital platforms to sustain and amplify their causes, highlighting the intersection of technology and activism under COVID-19’s impact (Poulakidakos et al., 2023).

### 2.1 The role of COVID-19 in CLN’s inception

The catalyst for establishing CLN can be traced back to a series of pivotal events and realizations stemming from the participation of community members in global events on biodiversity. CLN’s chairman states: “the realization that we faced common challenges across Southern African countries prompted our action. Our experiences, especially in interactions with the Convention on International Trade in Endangered Species of Wild Fauna and Flora (CITES), highlighted a significant gap: the lack of local community representation in its processes. This insight, gained during divided meetings at CITES, underscored the necessity for a united front to amplify local voices” (Lubilo, 2024). According to the network’s secretary-general, a Conference of the Parties (COP) held in Geneva in 2019 and subsequent meetings affected by the COVID-19 pandemic, highlighted the challenges they faced in advocating for the interests of the Indigenous peoples of Southern Africa (Louis, 2024).

In Namibia, the core issue wasn’t about the government permitting foreigners to purchase land for industrial or agricultural uses, often referred to as land grabs. The real challenge stemmed from land invasions, driven by drought conditions. People, in their quest for sustenance for their cattle and goats, would illegally encroach on lands designated for wildlife due to the dire need for food resources. During the COVID-19 pandemic, a shift in land use patterns was observed, as people, cut off from their usual food supplies, exerted increased pressure on the environment. This involved deforestation and other activities detrimental to the ecosystem, all in an effort to secure their livelihood and food security. Thus, the formation of CLN coincided with a period marked by a multitude of challenges, some of which diverged significantly from the movement’s initial planned focus, highlighting the broad spectrum of issues and struggles faced by these communities.

But what we saw during COVID was that the land uses changed because people did not have access to food, so they just started putting more pressure on the environment, cutting forests, you know, all of this for them just to make a living, to address their own food security. So that's what they were doing. The environment suffered because of what was happening. So, it was a very difficult time that we formed CLN because there were all these issues and challenges that came through, and some of them were not even related to the issues that we initially thought we were trying to address. – Louis, 2024

The choice to launch CLN amid COVID-19, despite the widespread assumption that the pandemic would severely limit organizational activities, presents an intriguing case. Opening CLN amidst the pandemic was driven more by necessity (urgency) than by a deliberate plan (strategy). Its founders chose to proceed during this difficult period rather than postponing their efforts until after COVID, driven by concerns that the issues they aimed to tackle would only worsen if left unaddressed. The pandemic, for all its hardships, provided a unique opportunity for reflection and planning. With the slowdown in regular activities, they found the quietude necessary to deeply consider the foundation of their organization. This period of reduced distractions allowed them to concentrate fully on establishing the institution and addressing the pressing issues at hand. Remarkably, COVID-19 became a catalyst for their initiative, facilitating active engagement, discussions, and debates within their network, particularly through WhatsApp groups, highlighting the unexpected opportunities that arose from the pandemic for collective action.

Against COVID’s uncertainties and subsequent expectations, CLN’s founding members managed to successfully establish an activist network. Scholars describe the observed phenomenon as follows: “the tendency to self-categorize in terms of the salient group is elevated when uncertainty is high and intergroup comparisons are pronounced” (Abrams et al., 2021). Consequently, in the face of an unstable external situation like the COVID-19 pandemic, people’s inclination toward seeking a positive group identity intensifies as a means to mitigate uncertainty (Shin et al., 2022).

The emergence of CLN during the pandemic further illustrates the resilience and determination of Southern Africa’s rural communities. Despite the challenges posed by the pandemic, which saw many organizations and projects falter, the formation of CLN stands as a testament to their collective strength. One would observe that such a bold move underscored the network’s capacity to persist and adapt, maintaining communication and coordination against odds. At first glance, the action of CLN’s founders might be seen as audacious. However, from a rural standpoint, it was merely business as usual—having to routinely undertake initiatives to navigate societal and modern pressures, a testament to their historical and enduring spirit of resilience. Their resolve was crucial, especially in the face of external pressures and challenges, including intimidation and attempts to co-opt the movement for other agendas.

We couldn't die. We resisted to die. Our success in founding CLN amidst such adversities reflects a broader narrative of resilience and autonomy. The establishment of CLN during a global crisis not only signifies our ability to overcome immediate threats but also reinforces our commitment to self-led advocacy and the pursuit of shared goals across communities. That just shows how strong our communities are. We were determined. We continued to interact, to communicate in the midst of all the threats of COVID. We were intimidated. Others didn't want CLN to be born. Others wanted CLN to be their network. Others wanted CLN to be a conduit for raising money. We told them, don't worry, we'll meet on our own needs. And once we met, we acted and survived. – R

### 2.2 Grassroots resilience amid pandemic challenges

CLN has challenged the widespread notion that COVID’s restrictions would obstruct the development of activist networks and movements. The inferred question thus is: what were the pandemic-imposed restrictions crafted for, with what objectives in mind, and who were the intended audience? The primary goal of COVID-19 restrictions was not to curb organizational activities, as the latter rather emerged as an unintended side effect of the pandemic itself. Rural communities likely perceived these pandemic-imposed limitations to be just that, limitations imposed on those directly impacted by the pandemic, not on them. Not only did they perceive these measures merely as constraints aimed at controlling the pandemic, not directly at them; but also saw them as primarily targeting urban populations and spaces, which were directly affected by the pandemic, rather than applying to their own circumstances. There were no such restrictions that they had not already been accustomed to. This perspective may stem from a familiarity with being excluded from national and global decision-making processes and operating with limited information about issues affecting them directly, an idea encompassed in Dolšak and Ostrom’s notion of “bounded rationality” (Dolšak and Ostrom, 2003). Consequently, this lack of comprehensive knowledge about the pandemic, rather than paralyzing them with fear or indecision, may have shielded them from the anxiety and hesitation that often accompany overexposure to information. Empowered by this relative ignorance, they were able to continue pursuing their objectives without undue concern.

For me, the formation of CLN in 2020 stands out as a pivotal moment. From our inception, we immediately began to disseminate precise data and figures, challenging previously accepted inaccuracies and fallacies. During the pandemic in 2022, we tackled the UK [anti-trophy hunting] bill, followed by efforts in Brussels. Travel restrictions and health safety measures due to COVID-19 made these endeavors particularly challenging, with stringent testing requirements across countries. Before the formal establishment of CLN as a coalition around 2014-15, we had already engaged in two significant meetings in the US, laying the groundwork for our future activities. – R

The experience of operating during COVID-19 has underscored the feasibility of remote collaboration, reducing the need for physical meetings and travel. This has opened up new avenues for efficient operation and communication via platforms like WhatsApp and Skype, further supporting their goal of financial sustainability by lowering operational costs.

COVID has taught us good lessons. Look here, we are making progress. We are discussing on WhatsApp, skype. I didn't need to fly to Florida to have this interview, you didn’t need to fly to Zambia to look for me. Similarly, CLN shares its history with new members and the broader community through digital means, given the logistical and financial constraints of in-person meetings. Our primary mode of communication and engagement is a WhatsApp group, complemented by regular online meetings. This approach became especially crucial during the COVID-19 pandemic, allowing us to maintain continuity and interaction despite the circumstances. However, we acknowledge the difficulties posed by limited internet access in the rural areas where many of our community members are based, which presents ongoing challenges to our digital communication strategy. – R

COVID-19 restrictions varied widely across different countries and regions but generally included a range of measures intended to control the spread of the virus. These restrictions were implemented based on the severity of outbreaks and the advice of health experts. However, while implemented globally, the restrictions resonate with the lifestyle of rural people in unique ways, reflecting both similarities and inherent differences due to the rural context. Here’s how each of the restrictions aligns with or diverges from typical rural lifestyles (Table 2).

**Table 2:** COVID-19 restrictions and alignment with rural realities.

Common COVID-19 restrictions	Consequences of pandemic restrictions	Alignment with rural realities
**Lockdowns**: Entire countries or specific areas were locked down, restricting movement except for essential reasons such as grocery shopping, medical needs, or essential work	**Social and Mental Health Issues**: Increased isolation and stress from lockdowns and restrictions contributed to mental health issues, including anxiety, depression, and loneliness	**Lockdowns** : Rural areas often have lower population densities, which naturally limits the frequency of close contact with others. Thus, the concept of staying within a confined area might not drastically change the day-to-day life of rural inhabitants, except for restrictions on market days or communal gatherings which are vital for their economic and social life
**Social Distancing**: Recommendations or mandates to maintain a certain distance (often 6 feet or about 2 meters) from others to prevent virus transmission	**Innovation and Adaptation**: Positive outcomes included accelerated adoption of technology, remote work, and innovations in healthcare and communication	**Social Distancing**: Social distancing is inherently practiced in rural settings due to the spread-out nature of homes and farms. However, this practice might have been more challenging to maintain during essential gatherings, such as at markets or community meetings, which are crucial for rural life
**Mask Mandates**: Requirements to wear face masks in public spaces, especially indoors or when social distancing could not be maintained	**Education Disruption**: Schools and universities were closed or moved to online learning, affecting the quality of education and social development for students	**Mask Mandates**: The requirement to wear masks in public spaces may have been a novel concept for many rural communities, especially in areas where access to healthcare and public health information is limited. Compliance would likely depend on the availability of masks and the extent of awareness efforts by local authorities or NGOs
**Travel Restrictions**: Limitations on international and sometimes domestic travel, including bans, quarantine requirements, and testing protocols	**Healthcare System Strain**: Non-COVID medical services were often postponed or limited to prioritize COVID-19 cases, impacting those with other health conditions	**Travel Restrictions**: While international travel restrictions might have had less direct impact on daily rural life, restrictions on domestic travel could significantly affect rural communities by limiting access to markets, healthcare, and other essential services not available locally
**Gathering Limits**: Restrictions on the size of gatherings, affecting events like weddings, funerals, religious services, and public gatherings	**Environmental Impact**: Temporary reductions in pollution and greenhouse gas emissions were observed during lockdowns, alongside changes in wildlife behavior	**Gathering Limits**: Limiting the size of gatherings could profoundly impact rural communities, where social, cultural, and religious gatherings play a central role in community life. Such restrictions might hinder communal practices, including agricultural, religious, and social events, which are essential for bonding and mutual support
**Closure of Public Spaces**: Public spaces such as parks, beaches, and recreational facilities were closed or had restricted access	**Inequality and Disparity**: The pandemic exacerbated existing inequalities, with marginalized and lower- income communities facing greater health and economic challenges	**Closure of Public Spaces**: In rural areas, public spaces like community centers, parks, and recreational facilities serve as crucial hubs for social interaction and community activities. Their closure would impact the social fabric of rural communities, although the effect might be mitigated by natural outdoor spaces that remain accessible for personal recreation
**Closure of Non-Essential Businesses**: Businesses deemed non-essential, including restaurants, bars, entertainment venues, and retail stores, were closed or had limited operations	**Economic Impact**: Significant economic downturns due to reduced consumer spending, travel, and business closures. This led to job losses, financial instability, and challenges for small businesses	**Closure of Non-Essential Businesses**: The impact of this restriction would vary significantly depending on the rural area’s reliance on local businesses for goods and services. In many rural communities, local shops, markets, and informal vendors are essential for daily life, making closures more disruptive. However, the definition of “non-essential” might differ, with a greater focus on agricultural and food-related businesses that are critical in rural economies

The term “limited operations” here refers to a situation where a business, organization, or system is functioning at a reduced capacity, due to various COVID-19 induced factors, such as resource constraints (e.g. limited availability of resources like staff, materials, or funds), external factors (e.g. supply chain disruptions, or regulatory restrictions), technical issues (problems with machinery, software, or infrastructure), or strategic decisions (deliberate choice to scale back operations temporarily for reasons like low demand, cost-saving or focusing on core activities).

Overall, while the implementation of COVID-19 restrictions had universal goals, their resonance with rural lifestyles reflects a blend of natural alignments due to lower population densities and significant challenges posed by the centrality of community gatherings and limited access to alternative services. For once, ironically, global measures inadvertently played in their favor.

### 2.3 Overview of the digital landscape in Southern Africa and its accessibility to indigenous populations

The digital landscape in Southern Africa, like much of the continent, has experienced significant growth over the past two decades. Increased access to mobile phones and the internet has transformed social, economic, and political engagement, including within indigenous communities (Foyet, 2024).

Many of our members are also on social media. WhatsApp groups, Facebook, we use it to disseminate information and see in it a quick way of communicating. For instance, if I'm attending a meeting in London, we make sure that we cover it up, like we provide live updates or summaries, so that our people, even those in remote villages with smartphone access, can stay informed and follow some of these proceedings. This method has proven to be one of the fastest ways to communicate and engage with our audience. – R

However, disparities in digital accessibility remain, with urban areas enjoying relatively higher connectivity than rural and remote communities where many indigenous people reside.

In rural areas, people need phones. And buying a smartphone, that's a huge cost. But even if you buy it and don’t know how to tweet, you can’t maximize your phone. – R

To be candid, digital literacy and access among rural communities are exceedingly low. These communities often lack both the means to access information technology and the knowledge required to navigate it. To bridge this gap, we frequently rely on younger community members to foster literacy. The situation varies significantly by country, with some countries faring better than others. For instance, South Africa boasts relatively high network access, where a considerable portion of the population owns smartphones. In contrast, countries like Malawi present a starkly different picture, with digital access being a privilege for a small minority. The disparity largely hinges on each country's economic status and GDP. – M

Furthermore, the digital divide exacerbates an unequal discourse. For instance, even though Namibia’s mobile subscriptions exceed its population at 2.75 million, only a third of these use their phones to access the internet (WeAreSocial, 2023). Most (92%) are pre-paid connections, which raises issues around data costs for accessing online information. Only a quarter of Namibians are active social media users with the most growth seen in Instagram (230,000 users) and Twitter (460,000 users); and only 35% of Facebook users are over 13 years of age (DataReportal, 2024). Most men use Twitter and LinkedIn, and women use Facebook and Instagram (NACSO, 2023).

Maintaining communication with rural communities on the ground is a critical aspect of indigenous digital activism; yet connectivity challenges significantly impede their efforts. For instance, when invited to a high-level international meeting, it’s essential for CLN to first consult with communities to understand and consolidate their viewpoints. Coordinating these discussions and disseminating the gathered information across the rural areas they serve can span several days. The logistical hurdles are steep, with some community members needing to travel extensive distances just to relay information to those without direct access to social media or smartphones. These obstacles are formidable in Africa, making their operations particularly challenging. When they request additional time, it’s not due to a lack of diligence or promptness on their part; rather, it’s a reflection of their commitment to overcoming these operational barriers as swiftly as possible.

For us CLN executives to have that communication with our communities is really a huge part of our work and these connectivity issues hamper a lot of our progress. […] We need to communicate with communities to have a consensus on the issues they want us to deliver. Reaching out to communities to arrange for these discussions and getting that information out to all the rural geographies we represent can take us days. For the few community members who have a smart phone that can access social media, they have to drive 100 kilometers to go and inform other community members located in remote places. So in Africa, these things are not easy. I can tell you these things are very difficult. It is not as if we have very good service providers when it comes to internet, we struggle a lot, and it's extremely expensive. Specific to rural zones, we don't have cables out there for communities to be on the same page as the world. That's why we always say to people, give me a week, give me two weeks. And there's a reason for that. We don’t ask for more time because we are slow or lazy, or that we don't respond to issues as soon, but rather because we try our best to be the fastest we can despite the operational limitations on the ground. – M

Access to data poses a significant challenge for community engagement, often requiring rural activists to individually cover data expenses to facilitate timely communication. Recent issues, such as undersea cable disruptions, have further complicated their efforts to connect with the crowd. The internet service in Southern Africa, particularly in rural areas, is neither widely accessible nor affordable, severely affecting activists’ ability to stay aligned with global conversations and promptly relay community consensus on various issues. This lack of infrastructure means that even for those few with smartphones, disseminating information necessitates long trips to reach other community members in remote locations, making the work of indigenous activists exceedingly difficult.

Despite these challenges, CLN leaders recognize that social media offers valuable opportunities for rapid information sharing, allowing them to disseminate facts and connect with key stakeholders more efficiently than ever before. This immediacy is a significant improvement from the past, where responses could take much longer. However, the reliability of these digital platforms remains a concern.

## 3 The activists’ perspective

### 3.1 Purpose of social media usage

From the activists’ viewpoint, social media serves as a contemporary method for disseminating information. Because the communities they represent have very limited access to such platforms, they utilize it as a means to reach an audience that would otherwise lack access to the authentic information these communities possess. They thus utilize the digital realm to reshape the landscape of activism, mobilize in innovative ways, educate, and confront the barriers to justice and equity.

Our goal is to utilize social media not only as a tool for broadcasting messages but also for engaging in meaningful interactions, ensuring our audience receives accurate information directly from us, either as an institution or as individuals dedicated to conservation efforts. This approach aims to prevent the distortion of facts that can occur when information is passed through multiple intermediaries, as had previously been the case with third parties (M). The idea is to make sure that our message reaches a bigger audience, because we know there are a lot of people on social media and it's the quickest way of getting information out there. – R

#### 3.1.1 Audience

CLN’s primary audience encompasses a diverse range of individuals and groups, notably the Western public, where misinformation often proliferates to and from. It varies depending on the issue at hand, and includes both supporters and opponents of hunting.

Specifically, for topics like pro-hunting advocacy, we've focused on influential policymakers, such as members of Congress, the House of Lords, the House of Commons, and the European Parliament. The purpose of sharing information on social media platforms is also to ensure wider visibility of our news and stories, which aids in shaping the public narrative around issues we are involved in. So our target audience is determined by the specific matter we are addressing. – R

It also includes conservation enthusiasts who rely on accurate data for decision-making, donors and the private sector seeking insights during their visits to conservation areas, public and international bodies invested in conservation and wildlife preservation, the local communities they represents, and allies. A key focus is on engaging youth, regarded as future custodians of our planet, who may lack the means to experience these regions firsthand. Additionally, researchers and scientists find their information invaluable for their studies. CLN also aims to educate students and the broader public, believing that awareness of biodiversity and wildlife issues is a universal responsibility that benefits our global community.

I think it's everybody's business to know what's going on in terms of biodiversity and wildlife issues and our communities. It's everyone's business because it is our world, it is our planet. The planet does not just belong to Africans. The continent might be, but we are linked and we are globally linked to the planet. And so the environment is everybody's business. Biodiversity is everybody's business. A good example is climate change. Even though northern countries are the highest polluters, we cannot sit here in sub Saharan Africa and think it's not our business. It is our business because we are impacted by what other people are doing within their businesses. And if they are warming the world, the world is warming on our side. We are burning on our side. So when we are burning on our side, it just becomes everybody's business. It's not just our business. – M

#### 3.1.2 Audience reach

CLN’s communication specialists handle this aspect. They employ various methods, including email distribution. They also use integrated social media networks, a process of linking together different social media profiles and platforms to create a cohesive online presence and ensure seamless sharing of content across various channels. This interconnectedness of their website and social media platforms facilitates and quickens information dissemination to a wide audience. Essentially, their strategy revolves around maximizing visibility by linking social media platforms to numerous other outlets, ensuring messages reach the largest possible audience. CLN also ensures that the people within the rural communities have access to the information.

Living alongside wildlife and within diverse ecosystems positions us as custodians of a significant portion of the world's biodiversity, with a quarter of it found on the African continent. In Southern Africa, particularly through CLN, we prioritize ensuring our community is well-informed for decision-making and stewardship of natural and wildlife resources. They serve as the initial source of information for us, through various channels like meetings, workshops, radio broadcasts, surveys, and inclusion in research projects. Radio is a particularly effective communication tool in the rural context, complemented by mobile phones. Despite weak network signals, satellite technology enables remote interactions, including the use of WhatsApp and social media for a dynamic exchange of information. This two-way communication strategy enhances our mutual understanding and engagement in conservation efforts. – M

#### 3.1.3 Communication channels and techniques

Apart from email distribution, CLN uses a diverse array of channels to communicate.

We actively manage a Twitter account, frequently posting updates to keep our followers and other netizens informed, alongside utilizing newspapers and WhatsApp groups as alternative communication channels. Additionally, we distribute information through letters and regularly update our website with new posts, leveraging various digital methods to disseminate information without the need for physical interaction. Living in an increasingly online world, we have a communications aid who provides us with weekly and monthly social media analytics reports. These reports help us monitor the reach and engagement of our online content, allowing us to identify our weakness areas. For example, if certain posts receive little interest, we can pinpoint where improvements are needed. This analytical approach aids us in understanding the extent of our online information's accessibility and engagement. – M

They ensure netizens have access to the information in the following ways:

Much of our outreach efforts are conducted via social media due to the logistical challenges of disseminating information in person about on-the-ground activities. In the countries we operate, designated focal points help spread this information further. This approach has significantly influenced our operational methods and shifted perspectives on effective communication strategies. Utilizing social media not only proves to be cost- effective for sharing information but also more impactful, aligning with contemporary communication practices widely adopted by our audience. While there are associated costs, they are markedly lower than those incurred through traditional, physical means of information dissemination. – M

Besides WhatsApp and Twitter, we also utilize Facebook and have an official website. We haven't ventured into LinkedIn or Instagram as of yet. Additionally, YouTube has served as a platform for some of our promotional efforts. When asked about the most effective platform, I'd say Twitter stands out because it allows us to reach a broader audience, including not just our followers but also their networks. WhatsApp, on the other hand, is excellent for swift communication and fostering a sense of community within closed groups, where members can quickly discuss and provide feedback on various topics. – R

According to CLN’s representatives, the frequency of the organization’s posts and shares directly influences its visibility; the more they disseminate, the greater is their visibility. The latter allows them to form partnerships that play a vital role in further extending their reach. Aware of their limitations in covering all platforms or engaging every audience, they focus on circulating information and factual updates from the ground, keeping stakeholders informed about ongoing developments.

#### 3.1.4 Social media impact on movement’s goals

There have been numerous occasions where CLN’s social media presence was pivotal. Specifically, topics such as trophy hunting bans, the “30 by 30” conservation initiative, and human- wildlife conflict have seen significant engagement. They have also been involved in various broadcasts that are available on platforms like YouTube. These topics, among others, have greatly been influenced by and benefitted from their active social media campaigns.

Last year, the British Parliament introduced a Trophy-Hunting ban bill. In response, we launched a campaign, reaching out to our communities in countries (Namibia, Botswana, Zambia, Mozambique, South Africa, Tanzania, Angola) directly affected by it. Our efforts included composing and dispatching 650 letters to MPs in the UK’s Houses of Lords and Commons. Also, we informed the British public about these letters through publications, encouraging them to inquire with their MPs about receipt. We shared some of these letters with various media outlets to broaden the campaign’s visibility. Through these diverse strategies, our campaign successfully stalled the bill’s progression through Parliament. Within the countries where CLN is active, certain countries may not depend on CLN for addressing some of their rural challenges. CLN typically focuses its efforts on supporting countries with communities that specifically need and require its assistance. – M.

#### 3.1.5 Engagement with audience

CLN’s engagement with audience has been shaped by several factors, including financial constraints. Digital platforms, notably WhatsApp, Twitter, Zoom, and Teams, allow for widespread engagement and the conduct of nearly all their meetings online due to budget/logistical limitations.

Occasionally, we come across news from institutions or anti-hunting advocates. We seize this information and disseminate it through our WhatsApp groups, a platform that suits our current budget and proves to be highly effective. Our community groups, accessible to those of our members who can join, have become hubs for sharing and scrutinizing this information, allowing members to correct any inaccuracies. This process not only facilitates the distribution of news feeds but also supports active discussion and feedback. In addition to social sharing, we distribute documents and announcements through WhatsApp and email, inviting community feedback and fostering dialog. WhatsApp also enables us to plan meetings and organize debates on contentious issues to enhance our engagement and collective understanding. Currently, virtually all (99%) of our meetings are conducted online, primarily due to funding constraints and the logistical challenges of in- person gatherings. The digital tools that prove most effective for our communication needs, ranked from most to least impactful, include WhatsApp, Twitter, and Instagram for broader engagement, while Zoom and Teams are our go-to platforms for virtual meetings. These tools have become indispensable in our operations. – M

Example:

When we receive news about an impending trophy ban bill, we immediately strategize our response while staying in close contact with allies who keep us informed about the latest developments, whether they're occurring in the US, UK, Belgium, or elsewhere. Determining the timeline for conveying the bill's details to our community members and gathering their feedback for legislators is critical, sometimes necessitating physical meetings with communities for clear explanation. Upon learning from our allies when the bill is expected to be passed, we utilize this timeline to strategize our forthcoming actions, which might include engaging with communities directly, and drafting and distributing letters among other initiatives. WhatsApp significantly accelerates our communication, allowing us to respond swiftly to urgent matters. Following a consensus on WhatsApp, we then move the discussion to Twitter to spark public debate and further the conversation and our advocacy efforts. Through social media, CLN can announce updates, stir discussions, foster dialog, and forge alliances, leading to numerous partnerships. Social media is pivotal for organizing events, debates, meetings, and conferences that enable us to reach our extensive network without the need for physical gatherings, thus keeping our vast audience informed about our latest endeavors. – M

## 4 Perceptions of the audiences’ perspective

### 4.1 Social media’s role in connecting Southern African indigenous movements with the public

From the perspective of the public and audiences, social media serves as a bridge connecting them with social movements, particularly those centered around indigenous communities in Southern Africa. Upon the establishment of CLN, the response was overwhelmingly positive, as people appreciated having a structured organization ready to provide answers with authentic African perspectives, directly from Africans, rather than through intermediaries. This direct line of communication was well-received, though not by the intermediaries previously representing these communities. They were less enthused about CLN’s discovery and effective use of social media for outreach.

When we started setting up CLN, people were just happy to finally have an organized group of people that can respond to some of the questions they had and from which they wanted African perspectives directly from Africans and not through third parties. So for us, that was a really good beginning and people really like it, that they could directly communicate with us. However, the third parties that used to speak on our behalf did not like it that we had discovered that social media is a very good means of communicating. – M

However, the story is different for audiences in rural areas:

For the rural communities we advocate for, their understanding is largely informed by our reports, necessitating our role in educating them due to the lack of access to phones and a general unfamiliarity with social media among many. Despite our efforts to demystify social media, the concept remains elusive for some of them. They recognize that we have a method to relay their voices to the necessary parties, but the mechanics of this process may be beyond their grasp. However, they grasp the essence—that their messages are being delivered to the intended recipients. – M.

### 4.2 Audience engagement

In the real world, CLN garners respect and attention for the visible impact of their work on the ground. This is not to say that the digital sphere lacks engagement or support. Many online followers are incredibly supportive of their efforts. When asked how the attention span of both the real and virtual public impacts CLN, indigenous activists argued that while social media opens doors to connect with a global audience, offering significant positive potential, it also exposes them to the fickle and irrational character of social media users (Shin and Ognyanova, 2022) and the negativity from individuals with their own agendas. They maintained that direct interactions in the physical world allow for meaningful conversations and the opportunity to demonstrate their work firsthand, providing a stark contrast to the mixed experiences encountered online. This distinction highlights the value of both realms while acknowledging the unique challenges and benefits they present.

On one hand, the audience’s engagement is evaluated through social media metrics:

Based on the volume of inquiries we receive, it’s clear that our audience is engaging with our content on social media. This engagement often leads to follow-up meetings and published articles, indicating that people are learning about CLN and our activities through platforms like Twitter, WhatsApp, Facebook, and our website, to which we actively respond. – M.

On the other hand, animal rights supporters within the audience demonstrate skepticism toward CLN’s digital activism efforts, finding it hard to believe that communities can articulate and defend their own interests.

I was on Twitter when somebody requested that rural individuals come and type themselves. I replied that because they don't have access to this gadget, they have elected me to voice their concerns to the online community. But this person wouldn’t believe me and kept saying it is not true. Often, when advocating for community issues, critics dismiss our efforts with, “Oh, it's not you behind this work. It's somebody else that is doing it for you”. – M

Online discussions can be fraught with cynicism, where detractors, possibly motivated by their own agendas, use intimidation to shift focus away from pressing issues. Community leaders believe that these netizens’ critics are merely performed in exchange for incentives, from antagonists who want to perpetuate the pervasive stereotype that Africans lack the capability to utilize modern technology. According to them, this stance reflects not just disbelief but also discrimination and racism within social media circles, revealing a deeper layer of bias that extends beyond simple skepticism.

Often, the people online are filled with third parties that are challenging you on purpose in public using all sorts of intimidation techniques to distract the public away from the real issues at hand. Maybe they are paid up by handouts, that’s why they are speaking for them. There’s still that conception that Africans cannot use phones. And that’s why I’m even saying, even within social media, people discriminate and they are racist. – M

When faced with skeptics who doubt their efforts or claim they are not genuinely involved, community leaders strive to provide ample information to those open to understanding.

People like that who do not believe us, we try and give as much information as we can. But you also find people that will never accept the truth of our work because they have an agenda. So those ones we just ignore. – M.

The two last sentences of the above statement bring in light a phenomenon known as selective behavior, where users surround themselves with information and perspectives that align with their existing beliefs. This tendency to seek out agreeable content over conflicting views, documented by scholars like Sears and Freedman (1967) or Iyengar and Hahn (2009), raises concerns about the polarization of users into distinct ideological groups. In this case, selective behavior is observed both from the audience, who may “never accept the truth” (selective perception and selective retention), and the activist, who may “ignore” those that faint understanding (selective attention and confirmation bias). This observation corresponds to Shin’s view of social media as “a space where users are enclosed in a media bubble of their own” (Shin, 2020). The ease with which individuals can ignore opposing viewpoints, through simple actions like following or unfollowing, has fueled the belief in the existence of highly partisan and isolated digital communities, often referred to as ideological echo chambers (Sunstein, 2018).

#### 4.2.1 Perceptions of the policymakers’ perspective

When asked about their perceptions of policymakers’ perspectives, community leaders’ responses implied that western policy makers had a great understanding of social media and proficiently made usage of it. The rise of social media has undeniably altered the landscape of policy influence and advocacy, demanding new strategies for engagement and response.

It's hard to say exactly how policymakers view our social media efforts, as their perspectives can vary widely. However, from our standpoint, we believe they receive our messages. Whether or not they actively engage with them is a different matter, but our main goal is to ensure our message is heard. We have observed instances where our content has sparked constructive discussions in legislative bodies like the House of Lords or House of Commons, indicating some level of impact. While not all policymakers may openly acknowledge the influence of our social media, there are definitely those who recognize and value the information we disseminate through these channels. – R

In contrast, they expressed that social media represents both a tool and a challenge in the realm of governance and policy formulation in Southern Africa.

Recently, during an activity involving the UK, participants easily received and engaged with our message through their digital devices, reflecting the ease with which Western politicians navigate social media. Conversely, in our regions, the digital gap poses a challenge, particularly for local politicians less accustomed to social media, prompting us to also disseminate information through newspapers, a medium they are more accustomed to consulting here. Our politicians seek to stay abreast of developments within the country, yet reaching them via social media can be difficult due to their varying familiarity with digital platforms. To bridge this gap, we engage in dual communication strategies. There really is a need for enhanced digital literacy and capacity building at the institutional level here. – R

In Namibia, for instance, there’s a government-led push for digital skill enhancement, including social media training for officials. This initiative is crucial, especially considering many politicians hail from rural backgrounds and may lack prior exposure to digital tools. As they transition into parliamentary roles, the need for comprehensive digital training becomes evident, despite their political acumen, to ensure effective communication in today’s digitally driven landscape.

Nevertheless, it’s crucial to remember that the success and impact of these initiatives largely hinge on the specific political environment and the nature of the country’s political setup. Regarding the influence of social media on national politicians and policymakers, it’s apparent that many are cautious about having their names and actions highlighted in public forums, possibly due to apprehension about public scrutiny. Often, those in charge of resources seek to gauge the public sentiment through the information shared by indigenous activists, making it a subtle yet complex form of influence. While it’s hard to quantify the exact impact, this experience underscores one of the many ways political figures may interact with social media content.

When it comes to how our national politicians and policy makers are influenced by what goes on social media, I think when you put things out there, politicians never really want to hear their names in a negative light in newspapers. So most of the time, the ones that handle resources will probably try and access information from me and then sense what is the general mood out there. So yeah, it is difficult. You cannot really tell. This is not the only method. I’m sure there are so many methods, but that’s the one thing that I have experienced. – Ma

#### 4.2.2 Conquering misinformation

Analyzing social media data, particularly around trophy hunting in the UK, revealed skewed numbers that nonetheless shed light on public perceptions. It became apparent that misinformation was influencing public opinion and decision-making on this issue. By correcting the narrative with factual information, indigenous activists observed a shift in the public’s stance. In their views, this experience highlighted the extent to which inaccurate data was skewing perceptions. Delving deeper, rural activists reported that they have realized that these misconceptions significantly influenced public decisions, underscoring the importance of providing accurate and truthful information to reshape understanding and viewpoints on conservation topics. To overcome misinformation, they conduct fact-checking via universities and trusted experts or researchers well-versed in the subject matter to verify the credibility of information. This approach ensures that the information they rely on and disseminate is both accurate and reliable, and not an instance of selective sharing (Shin and Thorson, 2017).

I normally will go back to universities and just make sure that this information is credible, or I will check it with somebody who I know can help me out in verifying the information. A lot of times it will be universities, or it will be researchers that I know are in this field, or people in statistics agencies. – M

To address misinformation and uphold its credibility, CLN actively shares accurate information across its social media channels; and responds proactively when encountering false information about the network or relevant issues within its region of operation. This response may include tweeting clarifications, publishing official statements on their platforms, or even writing formal letters to articulate their stance. Also, they engage with the media, participating in interviews to ensure their viewpoints are accurately represented and disseminated. This multifaceted approach allows them to correct misconceptions and maintain the integrity of their message.

When for instance, we know that there’s a misinformation about us or about certain things within our continent or within our sub region, we sometimes tweet about it, write a statement which we post on our social platforms so people can read that we have disputed. There are times when we have written letters to put our position out. Sometimes we have taken interviews with the media so that they can publish our responses. – Mo

Grounded in factual information, CLN’s approach responds to the demands of a category of social media users for concrete data and thorough analyses. Transitioning from initial emotive reactions to a more data-driven strategy, the network focuses on the specifics of inquiries and engages in meaningful dialog, steering clear of emotionally charged exchanges that could detract from the core issues. Their goal is to ensure discussions remain focused and informative, providing a clearer understanding of the subjects they advocate for.

When it comes to CLN, that's why we work on issues around facts. I think a lot of it at the beginning was very emotive. And so we have moved away from emotions to facts because those are the questions that people are asking […], you know, give us numbers, analyze these numbers and tell us what you have, you know, all these things make a lot of sense. So that's how we use some of the information by virtue of looking at what the question is that is being asked. We try to make our interactions as practical as we can without falling into the traps of those using emotions to sidetrack the conversation, and ensuring we are not diverting but rather still address the matter at hand and making sure that they're better informed about our realities. – Mo

When asked about what they think of social media content and how it impacts the long-term goals of CLN, the response tinted the immediacy and ephemeral characteristics of social media content. According to rural activists, focusing excessively on social media to counteract misinformation detracts significantly from their primary conservation objectives, hindering their ability to achieve set targets. This diversion not only impacts their goals but also their financial support, as potential backers may withdraw their contributions due to the spread of negative information. Many online users, unaware of the consequences of their words, inadvertently cause harm while believing they are engaging in benign discourse. However, in African communities, unfounded accusations can lead to lasting issues, including a breakdown in trust and communication, which are challenging to rebuild.

In the long run, you have targets that you want to reach, but you cannot reach those targets because you are spending time on one activity, which is social media, addressing negative and misinformed information, spending your time on that. So that impacts our goals. That impacts also our money because some people might even withdraw in terms of supporting our communities. So all this information can have long term impacts in terms of the work that we are doing. A lot of times, to be honest, a lot of these netizens think that they do good, but they do harm unknowingly. Because for them it’s a game of words. It’s a game of playing with words. But for us, as Africans, you know, once you say something about somebody that is not true, it creates a problem that people then start closing up and they do not want to talk to you anymore. They do not want to engage with you around these issues and you lose trust. And to build up that trust, it’s very difficult. – M

#### 4.2.3 Traditional and emerging power structures

Radio remains the preferred medium among community members in Southern Africa. It is particularly valuable for reaching rural communities due to its accessibility in local languages. While rural activists occasionally make television appearances for interviews, they utilize a broad array of channels to spread their message effectively.

Radio is the popular one, especially for our members. We also sometimes go on television if we are being asked for interviews and so forth. We use everything that we can to be able to get the message across. But radio, especially for rural communities, is very popular, especially because it allows them to access information in local languages. - M Also, opportunities to be interviewed or to contribute to articles allow us to influence and participate in broader conversations. – Ro

In addition to mainstream media, the use of arts, particularly in educational settings like schools, plays a significant role in the communication strategy of indigenous activist groups such as CLN. They also venture beyond traditional conservation channels, engaging with platforms unrelated to conservation to broaden their outreach.

We have embraced drama as a tool to share our message and engage with the public. – Ro

We also participate in interviews and are featured in documentary broadcasts, as avenues to extend our reach. […] it does not necessarily have to be conservation platforms all the time. As I said at the beginning, this is everybody’s business, not just our business. Do you understand that? – M

As mentioned earlier, the task of conservation and of spreading environmental awareness is a shared responsibility, not solely indigenous. In the view of community leaders, it is a collective one that is not confined to CLN alone.

## 5 Communication infrastructure, platform design, censorship, and algorithms

### 5.1 Influence of formal institutions (elite) on information control and dissemination

There’s a consideration regarding who holds power over social platforms, primarily based in the West, which inherently influences message distribution. Rural activists have observed instances where platforms like Twitter have imposed bans or censored content, indicating their control over the narrative. Little is known about the logic of the platforms’ algorithms, prompting the need for algorithms audits (Shin and Valente, 2020). They stress that African engineers have not yet developed a social media platform to have direct control over its own content distribution, thus forcing Africans to navigate within the systems established by others.

The narrative is that those who control these social platforms are the same people from the West. So they can control the way the message gets disseminated. We have seen in some situations where twitter has banned people or censored information. Unfortunately us in Africa have not reached the level where we are able to have our own social platform that we can control. So we still have to play around with the colonialist’s tool, and in his arena, you know! – R

The influence of colonial history has skewed the technological landscape, limiting the advancements and contributions of other geographies to digital platforms (Tilly and Wood, 2015). In the views of rural activists, had history unfolded differently, without the setbacks of colonialism, perhaps Africans might have been pioneers in the development of digital platforms (Tilly and Wood, 2015), standing on equal footing with the rest of the world. “Unfortunately, our past not only shadows our present but also dictates how we navigate the digital domain, often compelling us to adopt a defensive stance or seek validation in ways that are foreign to our traditions” (Louis, 2024).

You know, if the scenario was that there was no colonialism to make us behind in terms of what we are today, if the scenario was normal, we would have developed a lot of these platforms on our own, and we would have been equal in terms of doing things. Unfortunately, the weight is not the same. We have been affected by our past, and our past is the one that now determines exactly what we do and how we move around some of our features, like we have to be apologetic for everything we do, which I think it’s absolutely nonsense. We have to do things because the West is doing it. And that is how social media has actually turned us up. – M

Those with ample resources, those who wield control over social media channels and financial assets, inherently possess greater influence. To illustrate, consider the scenario of implementing a trophy ban: proponents of the ban can swiftly mobilize and disseminate their message due to their extensive networks and rapid communication capabilities, outpacing the rural communities' ability to respond or disseminate their perspective to critical stakeholders in the UK or Africa who might oppose such initiatives. For rural voices, the challenge lies in the time-intensive process of gathering and conveying their collective stance, which includes securing data, undertaking lengthy journeys to reach remote communities, and formulating a unified message. Often, by the time these efforts come to fruition, key discussions or decisions have already advanced without their input, highlighting a significant discrepancy in communication speed and reach.

So what they do, they do it so fast, […] they have partnerships, it’s so quick. For us, voices of rural people at the other end of the spectrum, the waiting game is the one that is a problem. We need to get data to these communities, drive hundreds of kilometers, find these communities to be able to draft a concrete and inclusive message that reflects their voices in unison. This takes time. By the time the bill has gone through parliament for debate to take place, that information has not reached those ones that need to be reached to make those decisions and join the decision making table. That is a problem. – M

### 5.2 Virtual platform design in information control and dissemination

Moreover, according to rural activists, the very design of many social media platforms may reflect the values of their engineers, who may equally be opposed to trophy hunting, suggesting that their social biases might be embedded within the algorithms they build, thus affecting how the platforms operate and influence the content Africans encounter and interact with. Given that these platforms are not of African creation, but tools designed and controlled from outside, this can put African voices at a disadvantage. The issue of unequal access to digital infrastructure further exacerbates this disparity, limiting their ability to engage equally in the digital sphere. While part of the responsibility lies with African entities for not developing or investing sufficiently in digital infrastructure, it’s also clear that external forces exploit these gaps to their advantage, fully aware of the operational challenges within African governance structures. Community leaders assert that it is crucial for their governments to invest in digital infrastructure, to enhance their capacity for swift and effective communication and decision-making. This dual responsibility underscores the complex dynamics at play, where both internal and external factors contribute to the challenges indigenous activists face in ensuring their voices are heard.

When asked about ever being censored on social media, a community leader responds that their balanced and factual communication strategy has helped them navigate potential censorship challenges effectively:

We’ve never really been censored and the reason is simple. The information we share out there is truthful and lacks emotions. We just state the story as it is. We are not offensive, nor abusive, even in the strongest provocation by the West to try and undermine our rights, we have always been careful in terms of how we respond to them. That’s why I’ve been writing them letters. We’ve been calling them demanding for explanations and an opportunity to answer. Some of them have given us the audience, and some, no. But we have accepted because we also appreciate their diversity in terms of opinions, feelings, and so on. So for somebody to see an elephant being killed, it is a sin. But to a person like me who is used to seeing it and knows what is happening, I cannot be emotional because I know it is going to contribute to somebody’s survival. Is saving lives a sin too? – R

Another CLN leader’s response correlates with the preceding:

So what we usually do, we inform our focal points, for instance, that on this this day, please try and find a [phone] network that is suitable for you to communicate. It’s not automatic that they are sitting there with a smartphone and things will happen. It is not obvious to be able to reach them instantly at any moment, due to connectivity issues, so what we do is agree in advance with them on a day to be reached [meeting 3] so they take measures not to face any issue that day. So we make this agreement [meeting 4] days before the main meeting day [meeting 3], which we also schedule to hold days before the main deadline we gave ourselves [meeting 2] prior to the meeting with international actors and decision-makers [meeting 1]; I tell the focal point, can you make sure that you organize your members or your community to be part of that [meeting 3]? So that’s how we work. It’s not like everybody has a smartphone in the community and we will just call the meeting and it happens. You need to do preparations. You need to do work before that happens. I guess WhatsApp is easier because then it’s just on the phone and you do not need to create an account. But with Twitter and the other ones, it’s a bit more complex because you need to go and be creating accounts and other long processes. – M

### 5.3 Perceptions on data analytics and metrics

Rural activists perceive the current digital environment to be driven by subjective metrics that do not always accommodate their perspectives or validate their experiences without the need for external affirmation. They believe the emphasis on social media metrics, such as likes, forces them into a cycle of seeking approval rather than sharing authentic narratives. This shift toward both a validation-based and cancel-based culture contradicts some indigenous values, where the substance of their stories and the truths they hold are paramount, not the emotional reactions they provoke or the digital approval they garner. The understanding is that online engagement in digital indigenism or indigenous activism is not driven by the pursuit of social media likes or approval. Instead, their presence on the internet is dedicated to addressing issues and upholding their dignity, not merely to engage in the digital exchange of likes. They are guided by facts and a sense of correctness (Shin et al., 2017), focusing on concrete realities and practical concerns rather than emotions. This approach underscores a commitment to action grounded in the real-world implications of their efforts.

Now we have to go on social media to prove that we have a fact. We have facts, why should we go on social media to prove that? You know, we have to do this because there’s likes on social media. We do not go online for people to like us. No, we go there to rectify the issues and honor our dignity. We also do not go online to like other people’s stuff. But we go because there’s facts and there’s something that inform us this is the right thing to do. We do not go on emotions. We are not, for us, it’s not about emotions. For us, it’s about the realities, the practicalities on the ground. That’s how we operate. – Ma

In a world where digital engagement is often measured by emotional responses and quantitative metrics, indigenous activists find themselves compelled to participate in a system that values popularity over authenticity. This reality is at odds with their principles, which prioritize factual and grounded discussions over what they call “superficial metrics”. As they navigate this digital era, the challenge lies in asserting the authenticity of their experiences and the richness of their cultures, striving for a digital presence that reflects their true identities and values, not one shaped by the expectations and norms of a system that doesn't fully understand or appreciate their unique perspectives.

And now we have to go into a system that is about likes, that is very emotionally driven, where the majority of people that have access to the gadgets make decisions. And based on that, we need to look at our likes and function based on them and what social media is saying. That is so fake! […] if you want to address issues around conservation and people in Africa, and especially in Southern Africa, we want to be real, not fake. – S

Accordingly, community leaders and activists think data analytics provided by social media platforms are not adequate for measuring the impact of CLN's activities. They argue that the reach of the network’s activities, as quantified by analytics, fails to fully capture the essence of CLN's impact, given their significant on-ground work beyond digital platforms usage. Despite the omnipresence of social media, their commitment to direct fieldwork remains unchanged, necessitating their own methods of impact assessment. They are cautious about analytics, and sustain that global and trendy overemphasis on social media engagement inadvertently diverts their focus from essential field activities that directly benefit their communities, leading to a form of mental fatigue they termed “social media fatigue” (Rodriguez et al., 2021). In their views, trying to convey straightforward, practical information to an audience with preconceived notions is incredibly time-consuming and often feels like an endless loop. This effort detracts from valuable time that could be spent engaging with communities on critical issues such as poaching strategies or conservation tactics.

[…] Analytics don’t adequately measure the impact of CLN activities. Because, after all, we still have to go and do some work out there in the field. Our lives continue with social media or not. We still need to do our own impact measuring on our own. Actually, what it does is that it puts more pressure on us because we spend more time on social media doing things rather than going out into the field to […] do work that really matters for our communities. In my experience, I find it as a social media mental fatigue, sitting on a gadget talking to somebody trying to explain something that you feel they should be understanding because it's very practical and simple. But because they have an agenda, you go on and on and on and on and on. That takes my time away from sitting with the community in the rural area to look at issues that matter, like looking at issues around who's poaching here, looking at issues around who's doing this. How can we have a strategy for this? Rather than that, now I'm sitting underneath some tree for 2 or 3 hours trying to find some network that will not even work, or may work for 5 min if I am lucky. This is what I'm doing right now, trying to explain to you things that I think are right. It takes too much time away from things that we have to do positively with our communities. – M

### 5.4 Nexus between digital indigenism and lack of resources

Indigenous activists confide that if they possessed adequate funding, they would not need to rely on external assistance for managing conservation efforts, including dealing with what they view as lengthy and sometimes fruitless trophy hunting debates. The necessity for these discussions, from their standpoint as rural Africans, arises from a lack of resources. With sufficient funding, they would leverage their own platforms for advocacy and action, rather than appearing to seek charity. In their opinion, the core of the trophy hunting discourse, therefore, is not about conservation but rather about who holds the power and the resources to influence resource management. If rural people had control over their resources, there would not be a need to justify their positions on social media. This dependency underscores a broader challenge faced by rural Africa in asserting control over its resources and narrative, positioning them in an unfavorable light of needing external support to address their concerns and initiatives.

I wish we had the money to deal with issues around hunting ourselves. I wish we could do this trophy hunting institutionalization ourselves so that we do not have to go, I mean, if we had the money and the resources, do you think we will have these discussions as both of us Africans sitting here and having this conversation? No, we will not. We will not because we would have been using our own platforms to be able to do this, but now we look like beggars […]. That’s why I’m saying when you look at issues around trophy hunting, it’s not so much about trophy hunting is about who is powerful in having the resources and those ones that make the decisions around those resources. That’s what that trophy hunting talk is all about. If we had the money and we made that decision ourselves, we would not have to go on Twitter and social media to explain ourselves. But now we have to, because we are unequal in terms of those resources. Now we have to become beggars, asking money from people from outside to be able to do that. So that is the unfortunate issues around Africa and its resources. – M

### 5.5 Censorship and mental health

Although CLN has never been censored on social media, a member has observed instances where comments directed at CLN were deleted due to their racially charged nature or because they contained personal attacks. The member has also experienced retaliation at a personal level, having been the target of attacks on Twitter. As depicted in the testimony below, as a human being, unfounded accusations and negativity are factors that are likely to drain human energy and may impact their well-being. In the case of community leaders, this negativity extends to the communities they support. When communities hear allegations that they are dishonest or financially motivated in ways similar to their critics, it affects them too. Consequently, according to a community leader, unfounded accusations have a detrimental effect on the collective mental health of a community.

In terms of how I deal with that, emotionally or mentally, to be honest, I’m a human being. If somebody says things that I think it’s not true, it drains me. It affects me as a human being, but it also affects communities. Once I communicate this to [the communities] saying: “Oh, they are saying, you guys are not talking the truth, you are getting money from this and this, like we are.” It affects them. So, it has some negative impacts on us mentally. – M

When asked about what changes they would like to see in social media platforms to better support conservation objectives, indigenous activists alluded that while they may not have the power to alter social media designs directly, they can exert influence, particularly by engaging with engineers and those responsible for algorithm and platform development. CLN claims it has the capacity to draft letters to technology companies, highlighting the existence of communities that are currently, in its view, underserved by and disrespected on their platforms. Its intention is to call for a reconsideration in how these platforms are constructed, advocating for respect and acknowledgment of the diverse needs and realities of all users. The organization believes that by informing tech companies about the adverse effects their platforms can have, including issues related to the violation of prior informed consent, where communities are discussed or named without their agreement, it could spark a change, leading to legal action on international grounds for disrespecting community consent.

Community leaders in Southern Africa have a developed an adaptative approach to utilizing social media, prioritizing planned strategies while remaining open to spontaneous opportunities that arise to offer them new insights or valuable information. This multi-mode communication model is similar to what Li et al. refer to in their work as the “semi-public nature of communication” (Li et al., 2021).

They believe that not all content encountered on social media is detrimental, and that there are numerous positive aspects and resources that can be leveraged to their advantage. For example, they stay receptive to emerging research methodologies or innovative practices shared by researchers on social media and that can enhance their community engagement or information gathering processes. This adaptability allows them to incorporate beneficial findings and approaches that align with their goals, utilizing social media as a dynamic tool for improvement and learning.

We have a strategy in place. First the strategy, but sometimes things come up that are also new and that we need to sometimes use social media as a way of informing us. There might be some information that is needed, for instance, around some community aspects that we did not think about and not necessarily everything on social media is very negative. There’s a lot of things that are also very positive that we can use. So we use whatever is making sense to us. For instance, there has been some research methods, maybe that we are looking at. Some of the researchers told me that sometimes there are things that are coming up and that we can use for communities, maybe to gather information a little bit differently. So those are the things that we sometimes see are found. – M

## 6 Language

The limitations imposed by platforms like Twitter can be stifling. The essence of how indigenous people communicate, rich in nuance and depth, often does not translate well into the brevity demanded by social media, particularly when constrained by international languages. This compression of indigenous narratives does a disservice to the fullness of their stories, which are inherently more suited to oral traditions. As Africans, their strength lies in verbal storytelling, a skill honed over generations, passed down through gatherings around fires or within communal spaces, rather than through written text—a convention largely promoted by Western cultures. The African’s preference for speaking stems from a cultural heritage that values oral transmission of knowledge and stories, where their expressions find their most natural form. However, the current digital landscape, dominated by platforms that do not align with these traditions, challenges their preferred modes of communication. This misalignment extends to their values around privacy, further complicating their engagement with virtual and social media platforms.

Despite these challenges, there’s an acknowledgment that the creation of their own digital platforms, tailored to their communicative preferences, is within their reach, yet remains unexplored. This oversight is a shared responsibility. It highlights a pressing need for Africans to innovate in ways that resonate with their cultural practices, ensuring that their stories are shared on their terms.

Let me tell you, I don't like Twitter to be honest. You know, even language, the way we speak, the way we converse in terms of any language, the way we write, totally different. So if you are inhibiting me because, the English language or the French language is much more shorter, that tells you that I cannot tell my story. I have to tell my story in a very shorter version that you also will not understand if I explain what my story is. That is the thing. And why should I write? I want to speak. We are better speakers. We are orally as Africans much more better suited to tell our stories orally. Writing is something which is promoted by the West. We are good at using and storing our stories in our brains and then getting them out there. Africans in general aren’t big on writing. But when you ask me to speak, it's something that comes naturally. And it's not about the personality here, I just know as an African, our stories from the beginning is that we listen, we sit around the fire, wherever in our kitchens or whatever it is depends in Africa where you are, and things are being explained to us orally. We don't sit like this and tell our stories. No, we converse. We have environments, we have platforms where we express ourselves when we want to express ourselves. And unfortunately, we have been forced to converse our issues on platforms that we don't feel comfortable with. I don't feel comfortable with Twitter. I don't feel comfortable with Instagram. We Africans are very private people. Seriously, we are very private people. When we want to speak, there are platforms, there are ways that we do things. We are forced by the Western world, and even our young people are forced to start taking up a culture that we don't normally have to take up. But I must come back to us and say we can actually do these platforms if we want to. But we have not done that. So part of it is our fault. – Ma

Misinterpretations and language on social media can lead to significant misunderstandings affecting CLN’s credibility and relationships. For instance, an incident involving a researcher in Namibia demonstrated how casually used words could ignite anger within communities, jeopardizing his work and necessitating extensive efforts to mend the situation. Such scenarios underscore the potent impact of words and the critical importance of clear, respectful communication. They reveal the complexities of managing digital discourse and the vital need to prioritize direct, positive engagement with our communities over navigating the volatile landscape of social media.

I have even seen that sometimes the problem is the use of words. Language is different on social media. What you say there and how you play around with words might mean something totally different for CLN and its members, and that can create a lot of mistrust between that particular person and you. I remember there was a researcher who was doing some work in Namibia, and then he said something to the community and they got very angry and said he should not come to the community anymore. That nearly affected his work. We had to do some damage control because that’s not what he meant, but because he was playing around with words, communities took it seriously. So it took us back to spend time now to convince the communities that this person should come back and finish his paper. He did not mean it in that way, but because he used this word, communities felt he meant it. So some of these things happen quite a lot sometimes, you know. – M

When asked about how they think the user interface and usability of social media platforms can improve the engagement of participants in CLN, one of its leaders said that CLN’s primary goal is to uplift and integrate indigenous knowledge systems which significantly shape their communication styles and their desire for recognition and acceptance. The leader proposed that such systems could greatly contribute to balancing the disparities in information dissemination that they often see from western perspectives.

## 7 Discussion

Conservation has famously been described as a ‘crisis discipline’ where decisions often have to be made on the basis of limited, incomplete, or absent data. Such shortfalls are especially acute in the tropics, which host the majority of the world’s biodiversity but which contain regions where scientific capacity is extremely limited. While social media offers a powerful tool for raising awareness and mobilizing support, translating online activism into concrete policy changes and real-world impact remains a significant hurdle. Moreover, it has become trivial to point out the digital divide’s profound implications on rural and sustainable development. The effectiveness of social media in achieving the goals of social movements, particularly in the context of indigenous communities in Southern Africa, can be analyzed by considering the perspectives of activists, the public, and policymakers. Each group interacts with social media differently, views technology distinctly, and their objectives and outcomes provide a nuanced understanding of their impact. Therefore, we contend that media movements are complex and varied, not just driven by straightforward oppositions or tensions. They are made of various interlinked components that contribute to their formation, structure, actions, spread and impact. These components are encapsulated under five factors, which we call the “interactive five,” each of which has at their core, “culture” as the common denominator.

### 7.1 The interactive five as nodes of media movements

Contemporary, or media movements, which encompass various forms of media activism, social media campaigns, and the broader dynamics of how information circulates and influences public opinion, are deeply complex phenomena. They are not just about the simple transmission of messages or content from creators to audiences, but involve a complex interplay of components encapsulated in the interactive five that are:

- **Context**: The socio-political, economic, and cultural contexts within which contemporary movements arise and operate can dramatically influence their reception and effectiveness. Overlooking this aspect simplifies the dynamics of media movements, ignoring how context shapes the interpretation and impact of media messages.
- **Nonverbal cues**: Although nonverbal communication is more associated with personal interaction, in media movements, visual cues (like images, videos, the esthetic design of messages and the integration of cultural sensitivities) play a critical role in conveying facts, managing emotions, reinforcing messages, and engaging audiences. Simplifying media movements to just textual or explicit content misses the depth added by these elements.
- **Cultural influences**: Media movements often transcend local boundaries, interacting with and being reshaped by diverse cultural influences. Ignoring the role of culture in shaping the content, spread, and reception of media movements leads to a superficial understanding of their dynamics and potential for cross-cultural impact.
- **Interpersonal dynamics**: The interactions among individuals within a media movement, including creators, activists, participants, and audiences, influence its direction and vitality. These dynamics include collaboration, conflict, and negotiation of meanings, which are essential for understanding the movement’s evolution.
- **Feedback processes**: Media movements are characterized by continuous feedback loops between creators, content, participants, and the broader public. This feedback can alter the movement’s trajectory, influence media production, and affect engagement strategies. Reductionist approaches that ignore these feedback processes fail to grasp the adaptive and responsive nature of media movements.

Additionally, the notion of “collective identity” has been the subject of lively discussion among Western academics, who have struggled to clearly define its boundaries (Flesher Fominaya, 2010; Polletta and Jasper, 2001) and critiqued its status as conventional wisdom (McDonald, 2002). However, in African contexts, the concept is well understood through the term “ujamaa” (familyhood), a principle articulated by former Tanzanian president Julius Nyerere. Ujamaa is founded on (a) the principle of human equality, (b) the ethical stance that domination or exploitation of one person by another is wrong, and (c) the universal aspiration for an individual to live freely and with dignity, in a state of peace with their community (Nyerere, 1969). This concept emanates from the core communal belief that the wealth generated by a community should be shared among its people, preventing any individual from accumulating wealth to such an extent that it enables them to enforce unequal, exploitative, or oppressive conditions on others (Karenga, 1984).

Although there is variation in how digital “collective identity” is defined, its value has been emphatically reasserted as a concept that remains instrumental in deepening our understanding of social movements (Flesher Fominaya, 2010). In Trere’s view, collective identity is, at its most basic level, a shared sense of “we-ness” and “collective agency” (Snow, 2001; Treré, 2019). It embodies a shared feeling of belonging, what Italian sociologist Melucci (1995) described as a collective and interactive consciousness formed by individuals engaged in shaping the direction of their actions within the context of both opportunities and limitations. It’s seen as a dynamic process, with scholars interested in its use focusing on the role of media technologies in spreading symbols and connecting individuals. If this is ujamaa, what then is digital ujamaa?

### 7.2 The digital ujamaa

The specific exploration of the culture factor in online activism is markedly underrepresented in academic literature. This oversight is notable, especially considering how the culture factor shapes social activism, through new entrants such as indigenous activists.

The digital collective identity or digital ujamaa refers to a shared sense of belonging and collective consciousness that is formed, maintained, and expressed through digital platforms and technologies. It emerges from the interactions of individuals online who are engaged in shaping the direction of their actions within the vast array of opportunities and constraints presented by the digital environment. This emerging form of collective identity leverages the internet and social media to circulate symbols, ideas, and values, connecting people across geographical and temporal boundaries. It is characterized by its fluidity and the ease with which it can adapt and evolve, thanks to the rapid dissemination of information and the dynamic nature of online communities. It encompasses the ways in which groups come together around common interests, causes, or goals on digital platforms, forming communities whose identities are shaped by shared digital experiences, communication practices, and the collective negotiation of meaning and purpose within the digital sphere. The concept relies on the critical role of communication in the formation and sustenance of collective identities within social movements, highlighting the necessity of “continuous act of recognizing and being recognized that implies a noticeable flow of information between social movement actors and the environment within which they act “(Treré, 2019).

### 7.3 Impact of data analytics on the retro-evolution of social movement studies

The vast amount of data generated by activists’ online actions has led to a computational turn in the study of social movements and media (Tufecki, 2014), marked by an increased use of quantitative methods to analyze large datasets from social media related to protests. However, this emphasis on big data analytics is shifting the attention from the cultural, social, and political contexts of protests (Kavada, 2012; del Mar Gálvez-Rodríguez et al., 2018; Klinger and Svensson, 2015)—how collective identity is formed and maintained—back to instrumental and functional aspects of social movements (Earl et al., 2014; Earl, 2015; Earl and Garrett, 2020; Earl et al., 2022).

According to Trere, this gradual detachment from the dimension of collective identity goes hand-in-hand with the progressive disregard for internal communication dynamics. Scholars are observably shifting the attention of their research from the “backstage” of digital activism, notably day-to-day communication practices that sustain social movements over time (i.e., Facebook chats and groups, email lists, WhatsApp messages, etc.) to the “frontstage” of digital activism, public-facing metrics measuring the impact of social movements communication with broader audiences (i.e., Twitter feeds, Facebook posts, YouTube videos, etc.) and their effects on organizational capabilities.

This shift in focus is too drastic. The focus on the later could have occurred without such a sudden and severe disinterest in the former. By neglecting day-to-day communication practices, “scholars fail to address them as fertile environments for the creation of expressive forms of communication, the exchanges of meaning, and the construction of a new sense of belonging” (Barassi, 2015a,b; Flesher Fominaya, 2015; Ganesh and Stohl, 2010; Treré, 2019). In Gerbaudo and Treré’s views, functionalist perspectives adopt an atomized view of society (Gerbaudo, 2015) that underestimates technology’s role in enabling activists to explore their multifaceted identities and exhibits limited interest in conceptualizing movements as collective spaces where media imaginaries and cultures are forged, shaped, nurtured and sustained (Gerbaudo and Treré, 2015; Treré, 2019).

As various researchers have illustrated (Benski et al., 2013; Flesher Fominaya, 2007; Romanos, 2013), digital media not only provide the organizational infrastructure in which protests and mobilizations are propelled and coordinated, but also constitute the communicative backbone where the expressive forms of communication that characterize the networked generations are manufactured, shared, and appropriated. – Treré, 2019

### 7.4 Trere’s fallacies of communicative reductionism

“Fallacies of communicative reductionism” refers to a critique of oversimplifying communication processes or the elements within them. This concept highlights the errors that can occur when communication is reduced to overly simplistic models, theories, or explanations that fail to capture the complexity, nuance, and contextual factors inherent in communication.

In the context of media-movement dynamics, reductionism highlights treating media technologies merely as transmitters of messages from sender to receiver without considering the roles of context, nonverbal cues, cultural influences, interpersonal dynamics, and feedback processes. Such a reductionist view could lead to misunderstandings about how media technologies work, are used within and by movements, and why they sometimes fail. The fallacies, therefore, might lead to ineffective communication strategies, misunderstandings, and conflicts, as they fail to account for the full breadth of factors that influence communication outcomes. Recognizing these fallacies can help individuals, organizations, scholars, and activists develop more nuanced and effective communication practices that effectively address the multifaceted and dynamic nature of the cyber-urban space (Lim, 2015) or human-machine interactions.

This argument is backed by Trere’s five fallacies of communicative reductionism. In his views, social movement activists “employ a complex, and often unpredictable hybrid of old and new, physical and digital, human and non-human, and corporate and alternative technologies to determine, develop and diffuse proposals to allies, multiple publics, dominant media and state actors; and at the same time, to cultivate, articulate and reproduce collective visions and identities” (Kidd, 2019).

In the exploration of media movement dynamics, the fallacy of *spatial dualism* artificially segregates digital/virtual/cyber from offline/physical/real activism. This perspective erroneously suggests that online activism operates in isolation, ignoring the interplay and mutual reinforcement between digital campaigns and ground-level actions. Such a division underestimates how online mobilization rather complements and amplify traditional forms of protest, leading to a more integrated and holistic view of activism where digital and physical realms synergistically enhance each other’s impact.

Complementing this, the *one-medium* fallacy simplifies the complex media environment by focusing on a singular technology or platform’s role in social movements. This narrow view overlooks the diverse media ecosystem activists engage with, where different technologies and platforms interact to create a multifaceted communicative landscape. By reducing the complexity of media activism to the functionalities of individual tools, this fallacy disregards how varied media forms collaborate within a broader media ecosystem to shape the narrative and effectiveness of social movements.

*Technological presentism* introduces another layer of critique, emphasizing an excessive focus on the newest technologies under the assumption of their inherent superiority for activism. This fallacy neglects the historical continuity of media practices and the enduring relevance of older, traditional media, which can still be highly impactful in contemporary activism, alongside emerging technologies. By chasing the latest advancements, there’s a risk of missing the powerful interplay between established and new media forms, essential for understanding and designing the complete media strategy of social movements and how it evolves over time.

Moreover, the *fallacy of technological visibility* points to an overreliance on quantifiable aspects of media engagement, such as social media metrics (number of social media posts, likes, or shares), at the expense of the deeper, often intangible, and unquantifiable elements of social movements. This fallacy reduces the complexity of activism to measurable outputs, sidelining the formation of collective identities, emotional engagement, and interpersonal dynamics within movements; and the symbolic dimensions of activism, solidarity and belonging. By focusing solely on what is easily counted, the fallacy misses the depth of movements’ influence and the broader societal changes they seek to inspire; and reduces the success and impact of social movements to metrics that can be misleading or insufficient for understanding the broader effects of activism.

Lastly, the *fallacy of alternativeness* uncritically embraces mainstream, corporate-owned social media platforms as inherently progressive tools for activism, without adequately considering their commercial interests, surveillance capacities, and potential for censorship. This fallacy overlooks the importance of exploring alternative media avenues that better align with the values and goals of social movements, and more closely reflect their ethos and objectives. By failing to scrutinize the implications of relying on corporate platforms, there’s a danger of shaping movements in ways that may not fully serve their intended purposes, emphasizing the need for a critical evaluation of media choices in activism.

Together, these fallacies stress the importance of a nuanced perspective that considers the spatial convergence, plurality, historical, qualitative, and alternative dimensions of media use within activism, challenging reductive narratives and encouraging a more comprehensive view of communicative complexity in social movements. As outlined by Emiliano Treré, they caution against simplistic or uncritical approaches to understanding complex media dynamics in social movements and utilization for social change (Table 3).

**Table 3:** Distinct aspects of Trere’s fallacies in the context of media movement dynamics.

Fallacy	Key difference	Focus area
Spatial dualism	Emphasizes the artificial separation between digital and physical activism	Challenges the notion of separate realms, advocating for an integrated view of activist efforts
One-medium fallacy	Oversimplifies by focusing on the impact of a single technology or platform	Critiques the narrow lens on media diversity, urging a broader view of the media ecosystem
Technological presentism	Prioritizes the latest technologies, overlooking the value of historical and traditional media	Warns against neglecting the enduring relevance and contribution of older media forms
Technological visibility	Overreliance on quantifiable measures of engagement, missing deeper elements of social movements	Highlights the need to consider unquantifiable aspects like emotional engagement and cultural identity
Fallacy of alternativeness	Uncritical acceptance of mainstream platforms, ignoring potential misalignments with movement values	Encourages scrutiny of platforms for better alignment with activism’s ethos and objectives

### 7.5 Implications for understanding media movements

A reductionist approach that views media movements merely as channels for message dissemination misses the rich, interactive, and context-dependent nature of how movements develop, spread, and influence. It leads to misunderstandings about the effectiveness of media campaigns, the role of participatory culture in shaping media content, and the potential for media to foster social change. Recognizing the complexity of media movements is crucial for effectively analyzing their dynamics, strategizing for social change, and understanding the multifaceted impact of media on society (Table 4).

**Table 4:** Comparing the roles and impacts of social movements, social media, and mainstream media.

Social movements	Social media (INFIs)	Mainstream media (FORIs)
Facilitates political expression of	Average citizen	Traditional power structures
Political discussion	Enabled between elite/mass (hor.)	From elite to mass (vert.)
Interaction b/w activists_public	Direct	Indirect/mediated
Rapid wide spread of info	Beyond geographical boundaries (global)	Within geographical boundaries (local)
Coverage particularity	Informality and freestyling—focus on engagement over accuracy	Journalistic standards/practices—focus on credibility and authoritative sources
Content	Broader range of voices and varies widely in reliability and depth	Restricted range of voices and binary
	Everyone (incl. Professionals + amateurs) and diverse (incl. Mainstream + new forms of content, personal opinions, viral news, memes, user experiences, etc.)	Professionally produced (few people) and curated news, feature articles, editorials, and reports focusing on a wide range of topics like politics, economy, society, and culture
Authoritative coverage	From actor’s perspective	From elite’s perspective
Questionable coverage	From audience’s lens	From mass’ lens
Shapes public opinion through	User-generated content/viral trends	Framing and the extent of coverage
Coping mechanism	Help individuals partly cope with issues	Do not help individuals cope with issues
Mobilization of public support	Ads/cheap to free	Ads/costly
	By highlighting issues and movements	
Organizational tool—eases	Organization/coordination through groups/pages based on common interests	
Building collective identity	Engagement in causes and participation in activism through networking attribute (friends, family, colleagues, neighbors, role models, etc.) Helps in creating a sense of community/ collective identity among movement participants, crucial for sustaining momentum and solidarity	
Narrative control	Offers users the ability to directly control and share their narratives	Subject to the editorial decisions and narrative framing of media outlets
Content control—subject to the Selective coverage of possibly marginalizing some issues	Editorial bias and narrative limitation of algorithms Issues based on trending topics, digital gerrymandering, censorship, personalization, targeted advertising	Editorial decisions and narrative framing of media outlets Issues based on breaking news, partisanship, higher bidder, cultural sensitivity, potentially marginalizing certain issues
Accessibility of information	Only reaches privileged minority/tech-savvy and internet users	Spread awareness to diverse demographic groups/non-internet/ICT users
Spread of misinformation	Rapid	Slow
Sensationalism	Exaggerated, provocative, or overly dramatic content to attract engagement (viewership, readership, clicks, likes, comments, shares, etc.), potentially distorting goals, and detracting from substantive discussion	Sensational stories that may not accurately represent reality, aiming at capturing attention or increasing ratings or sales, leading to a skewed perception of events, issues, or groups of people
Fragmentation	Create echo chambers, where individuals only engage with like-minded views, potentially leading to increased polarization and division	Echo chambers = printed media only distributed in specific areas such as airports, corporate lounges, offices, VIP settings (Forbes, etc.)
Surveillance and repression	Authorities use social media to identify and monitor activists and suppress dissent, posing a risk to participants’ safety and freedom	Authorities use selective reporting to suppress dissenting voices, limit visibility, impact reach and marginalize activists from public discourse
Media ownership	Controlled by tech corporations	Controlled by media corporations
Archival record	Permanent record of events, providing a historical account that can be referenced in the future—still subject to space limitation	Temporary access to physical records to due to environmental factors (space, weather, usage frequency, mobility, etc.)
Slacktivism	Online support that does not translate into offline/real-world action (sharing posts, signing online petitions, etc.), potentially undermining genuine activism	“Armchair activism” or “keyboard activism” = expressing support for a cause primarily through passive or minimal efforts (individuals voicing their opinions or support for causes through letters to the editor, call-in radio shows, opinion columns, etc.) without necessarily taking concrete steps to address the issues they are discussing

### 7.6 Algorithms

The advent of advanced digital, intelligent, and algorithm-driven technologies has led to assertions that the “human” is being profoundly “re-mediated.” For critics who find this transformation troubling, the argument is that humanity is now increasingly politically disaffected, as our very gestures, emotions, and routines are being digitally colonized by capitalist forces (Pedwell, 2019). The internet has proven to be no less immune to control than media systems that preceded it (Caplan and Boyd, 2016). In fact, surveillance, censorship, and retaliation for speech have arguably become easier as communication increasingly shifts online. For example, censorship has been proven to be heavily embedded in the development of Wikipedia and Baidu Baike corpuses (Yang and Roberts, 2021).

The customization of social media via converted algorithms, the growth of targeted advertising and social marketing and other means beyond the public’s awareness is raising concerns over the role of algorithms in dividing people rather than bringing them together over uncomfortable but necessary conversation (Pariser, 2011). Even though recent e-government policies (efforts by governments to use the internet and digital platforms to share information, engage with citizens, promote participatory democracy, and improve government services) aim to uphold Habermasian ideals by promoting open dialog, this proposed two-way communication between citizens and governments occurs on platforms owned and controlled by companies that operate with rules and interests that may not align with the principles of open information and public discourse. These algorithms are gamed by a minority of users who can prompt the companies to alter how they function, further complicating who defines the norms and values of a particular algorithm. This creates a tension between the goals of e-government policies to promote a democratic and open public sphere online and the reality of the private, often opaque control over the digital platforms where these interactions take place.

### 7.7 Definition and characteristics

According to Willson, “algorithms make things happen—they are designed to be executed and to bring about particular outcomes according to certain desires, needs and possibilities” (2016). Media theorist Tarleton Gillespie argues that today, algorithms have become the central logic that governs the flow of information upon which people rely (Gillespie, 2014). This growing influence means algorithms are now crucial in determining the content that becomes visible on digital platforms, thereby significantly impacting public discourse and the dissemination of knowledge. In essence, they function similarly to a newspaper editor, determining the relevance of information to users within specific contexts (Napoli, 2014; Zeynep, 2015). These invisible editors actively shape what content is made visible, guide what users see, read, and ultimately believe, making them pivotal in the formation of public opinion and knowledge acquisition. Algorithms are being used to automate content to obey local norms and laws, through a complex system of user feedback (flagging), human reviewers, and algorithms (Chen, 2014). Though automated, they are also used to classify, filter, and prioritize content based on values internal to the system, and the preferences and actions of users (Kacholia and Jin, 2013).

Despite the technical specificity associated with the term “algorithm”—a defined sequence of operations for calculations or problem-solving (…), encoded procedures for transforming input data into a desired output, based on specified calculations (Gillespie, 2014), and Trere’s culinary metaphor as a recipe for a meal (an endpoint), with a list of ingredients (variables) and a step-by-step description of a process that describes in a detailed order what needs to be done and at which exact point in time (codes) (Treré, 2018)—the concept has broadly permeated everyday discourse, often simplified to mean “the things computers do” (Caplan and Boyd, 2016). This simplification masks the complexity behind algorithms, which, contrary to the neutral and objective facade suggested by their mathematical underpinnings, are subject to the biases and subjectivities of their creators.

Algorithms also have societal and political ramifications, as they are not standalone technological units but are woven into complex networks of social, cultural, and political exchanges. Ignoring this “complex assemblage of people, machines and procedures” that constitute them (Gillespie, 2016) and therefore, the fact that they reflect specific worldviews (Postigo, 2014) may result in the obscuration of the agency of the people behind algorithms: in other words, the influence of those who create and manage algorithms has been underrated. Overlooking this perspective might hide the necessity of examining algorithms within the context of wider global socio-technical trends, such as the process of datafication, which refers to the conversion of social action into quantifiable online data.

Throughout the development process, engineers and designers make numerous decisions that reflect their own perspectives, biases, and priorities. Consequently, these subjective decisions become encoded into the algorithms, influencing how information is filtered, ranked, and presented to users. This discrepancy between the perceived neutrality of algorithms and their reality underscores the critical need to scrutinize the embedded biases within these systems, which shape how we interact with and understand the digital world.

Algorithms are illegible to most, revised frequently, and often depend on data that might be flawed in countless ways. Those underpinning major technologies are proprietary and companies are often invested in keeping them away from public eyes, both for competitive reasons and to minimize external manipulation. Most importantly, because most large systems involve algorithms that are connected to data and evolve based on input, studying them out of context does little to elucidate how one person’s search result or social media feed looks at any given time. – Caplan and Boyd, 2016

### 7.8 Application to conservation dynamics

The discussion on algorithms and their inherent biases has significant implications for the conservation industry and the ongoing debate between animal rights and human rights. In this context, algorithms can profoundly influence how information related to conservation efforts, animal rights, and human rights is curated, shared, and prioritized across digital platforms. This influence directly impacts public perception, awareness, and ultimately, the policy and decision-making processes in these areas.

- **Information curation and public perception**: Algorithms designed to filter and rank content skew public perception by highlighting certain types of stories or data over others. If an algorithm is biased toward more sensational or controversial aspects of the animal rights vs. human rights debate, it may prioritize content that exacerbates conflicts or oversimplifies complex issues, producing the negative effect of hindering mutual understanding and constructive dialog in the conservation community.
- **Bias in conservation content**: The biases of those programming algorithms can inadvertently prioritize certain conservation narratives or agendas over others. For example, if engineers hold a bias that animal rights should always supersede human rights, the algorithms they create might filter and present information in a way that supports this viewpoint, marginalizing alternative content that advocates for conservation approaches that are not animal-centric.
- **Influence on policy and advocacy**: The way information is ranked and presented can also influence advocacy efforts and policy formulation. Algorithms that favor content in favor of fortress conservation while downplaying arguments for human rights-based conservation shape public opinion and policy in ways that overlook open dialog and democratic participation. By overlooking the ethical imperative to consider the needs and rights of local and indigenous communities affected by mainstream conservation practices, these algorithms strengthen the status quo by fostering an exclusive and inequitable approach to environmental stewardship. A shift in the programing of these algorithms would not only promote a conservation model that respects and upholds human rights but also encourage a more sustainable and ethically grounded engagement with the natural world, ensuring that conservation efforts benefit both humanity and the ecosystem in a balanced manner.

It is thus necessary for those involved in conservation and digital platforms to critically examine and understand the biases encoded in algorithms. There is a need for balanced algorithms that fairly represent diverse viewpoints and the complex interplay between animal rights and human rights in conservation efforts. This includes developing systems that can recognize and mitigate their creators’ biases, ensuring a more equitable representation of information.

### 7.9 Implications for CLN

The complexities and opacities of algorithms, as inferred from the above description, have profound implications for CBNRM efforts and indigenous movements such as CLN. As part of its communication strategy, the latter relies on the dissemination of information and mobilization of support through digital platforms, which, as explained earlier, are significantly influenced by their underlying algorithms.

- **Visibility and representation**: Indigenous people and CBNRM initiatives often struggle for visibility in mainstream discourse. Algorithms that prioritize content based on popularity, engagement, or proprietary interests might marginalize these movements, making it harder for them to reach a broader audience. The frequent revisions and the opacity of these algorithms can further exacerbate this issue, as it becomes challenging for these groups to understand and navigate the digital landscape effectively to amplify their voices.
- **Bias and data flaws**: The reliance of algorithms on potentially flawed data can misrepresent indigenous movements and CBNRM efforts. For example, if the data feeding these algorithms does not accurately represent the priorities, needs, or voices of rural communities, their narratives may be underrepresented or distorted in digital spaces. This misrepresentation would in turn influence public perception and policy decisions, often to the detriment of these communities and their conservation efforts.
- **Proprietary algorithms and lack of transparency**: The proprietary nature of major technologies and the secrecy surrounding their algorithms mean that the public, including the indigenous populations represented by CLN have little insight into how their information is being filtered and presented to users. This lack of transparency makes it difficult for these movements to strategize their online presence effectively and advocate for fair representation and support of their causes.
- **Contextual understanding and individualized experiences**: The individualized nature of search results and social media feeds, shaped by algorithms, means that information about CLN, indigenous rights, or even CBNRM may not reach individuals outside these interest groups. This personalized approach to information dissemination can create echo chambers, where people are only exposed to content that aligns with their existing beliefs and interests, further isolating indigenous movements from potential allies and support.

These implications highlight the urgency of improving algorithmic governance, particularly in how algorithms affect marginalized communities and their access to digital platforms. For CLN, addressing these challenges would require advocacy for more transparent, equitable, and inclusive algorithmic practices. This includes pushing for algorithms that are responsive to the diversity of users’ interests and needs, ensuring that these movements can leverage digital platforms effectively to advance their causes. Collaboration between technologists, policymakers, and indigenous communities is crucial in developing digital environments that support, rather than hinder, the visibility and effectiveness of these important movements.

### 7.10 Automation and bots

Bots, defined by Phil Howard as “chunks of computer code that generate messages and replicate themselves,” are used to flood a particular topic and make it appear more popular, affecting ‘trending’ algorithms, which further push specific messages into public view. The tactics of using automation, such as bots, to manipulate algorithms and sway public opinion have significant implications for movements like CLN, who rely on digital platforms to raise awareness, mobilize support, and advocate for their rights and issues.

- **Drowning out indigenous voices**: By flooding social media with irrelevant or opposing messages, bots make it more difficult for genuine posts from indigenous movements to be seen or heard. This “noise” can drown out important conversations, diluting the impact of indigenous advocacy and making it harder for these movements to gain the visibility and support they need.
- **Shifting public discourse**: Automated processes can quickly shift conversation topics, insert doubt, or create false equivalencies in conversations that people would rather not have. For indigenous movements, this means that the focus can be swiftly moved away from their concerns, or their narratives can be challenged or undermined by artificially amplified opposing viewpoints. This tactic can weaken the clarity and strength of the messages being conveyed by indigenous groups.
- **Undermining credibility and trust**: The use of bots to inflate follower counts can distort perceptions of credibility and authority on social media platforms. For indigenous movements, this creates an uneven playing field where their authentic engagement and support might be overshadowed by entities with artificially inflated follower counts. This disparity can affect the perceived legitimacy and impact of their campaigns.
- **Influencing algorithmic prioritization**: Since algorithms may prioritize content from users with higher follower counts, entities using bots to boost their follower numbers can unfairly increase their visibility and influence. This means that messages from indigenous movements, which are less likely to engage in such manipulative practices, might not receive the algorithmic visibility or prioritization they deserve, limiting their reach and effectiveness.

## 8 Conclusion

The interplay between digital platforms and indigenous social movements marks a transformative era in activism, particularly in Southern Africa. Social media remains a powerful tool in the arsenal of social movements, with its effectiveness contingent upon the interplay between activists, the public, and decision makers. This paper has provided an examination of the role of Covid 19 and social media in indigenous activism in Southern Africa, particularly through the lens of the Community Leaders Network (CLN). By examining CLN, this study has explored the dual role of digital platforms as facilitators of global connectivity and potential arenas for cultural conflict. Utilizing a mixed-methods approach that includes netnography, in-depth interviews, and theoretical frameworks like the counterpublics and the Technology-Media-Movements Complex (TMMC), the study elucidated the multifaceted ways social media supports and challenges indigenous movements. It equally discusses the complexities of digital activism, highlighting the transformative potential and hurdles that come with digital engagement in marginalized communities striving for social change and recognition; as well as the opportunities, pitfalls and implications the evolving landscape of digital indigenism presents for policy and community advocacy. Looking ahead, the future of social media in facilitating social change led by indigenous communities appears promising but complex. The challenge and opportunity lie in harnessing this tool in a way that respects the complexity of indigenous ecologies, digital environments and virtual imaginaries while striving for impactful and positive change.

One of the most significant findings of this research is the pivotal role that social media played in the formation and operation of the CLN. The onset of COVID-19 catalyzed the establishment of the network, as the pandemic-induced slowdown provided an unexpected opportunity for reflection and strategic planning. Despite the challenges posed by the pandemic, including travel restrictions and increased pressure on local environments, the CLN successfully leveraged digital platforms like WhatsApp to maintain communication, coordinate actions, and mobilize support across Southern Africa on pressing issues faced by indigenous populations, such as land rights disputes, environmental degradation, and socioeconomic marginalization. By sharing stories and disseminating information about rights abuses, the CLN has been able to engage a global audience and galvanize support for their causes.

It was also uncovered that informal indigenous institutions provide a solid foundation for collective action, enabling CBOs to leverage communal bonds in their advocacy and development efforts. CLN collaborates with community leaders and leverages traditional community solidarity to mobilize support for indigenous land rights and sovereignty. Similar to many CBOs, it advocates for sustainable resource management and conservation practices, often opposing extractive industries that threaten their territories. It faces and challenges external pressures resulting from globalization, and navigates legal systems imposed by nation-states that do not recognize indigenous governance structures or rights. Despite these challenges, CLN stands through the strength of its members’ share of a common purpose. The resurgence of indigenous movements, bolstered by global networks and social media, has amplified their voice and influence.

Another revelation verified that while social media is a powerful tool, it also comes with significant limitations. Indigenous activists find that social media solutions are misaligned with their contextual needs, citing concerns over platform constraints, privacy issues, cultural insensitivity, and superficial engagement metrics. These challenges underscore the need for social media innovations that resonate with indigenous cultural identities and ensure the authenticity of their narratives. The CLN’s ability to adapt to the constraints of the pandemic and continue its advocacy efforts is a testament to the resilience of Southern Africa’s rural communities. The network’s use of WhatsApp, X, and other digital platforms facilitated active engagement and collaboration, not only demonstrating the potential of digital tools to sustain social movements even in the face of significant external pressures, but also providing evidence that Indigenous peoples can also be technologically savvy, contrarily to the mainstream assumption. Nevertheless, the research underscores the disparities in digital accessibility within Southern Africa, particularly between urban and rural areas.

These technologies offer new tools for storytelling, fundraising, and virtual collaboration. Yet, several challenges loom. These include the risk of increasing digital surveillance, the commercialization of social media spaces, and the perpetuation of inequalities in digital access and literacy. To maximize the positive impact of social media on social change, there must be concerted efforts to bridge digital divides, protect online freedoms, and ensure that these digital spaces remain accessible and empowering for indigenous communities. Collaborations between technology companies, governments, NGOs, and indigenous groups could be key to creating an inclusive digital future that supports indigenous rights and amplifies their voices in the pursuit of justice and equity. By addressing the challenges of digital activism and the digital divide, this work acknowledges the tenacity and resilience of new conservation movements, who continue to (a) find innovative ways to engage their audiences and disseminate crucial information, (b) ensure the participation of marginalized communities in online and global environmental discourse and policy-making, and ultimately (c) contribute to more sustainable environmental outcomes, despite facing challenges such as geographical remoteness, inadequate transportation networks, poor ICT infrastructure and inconsistent access to internet, and digital literacy and linguistic barriers. Furthermore, social media algorithms and fake bots, increasingly flagged for disadvantaging marginalized groups – by reducing their visibility and perpetuating stereotypes, for examples – are significant barriers to achieving social justice and effective conservation that must be addressed.

Overall, the research provides valuable insights into the complex interplay of social media, activism, and indigenous movements in Southern Africa. It highlights the transformative potential of digital platforms while also calling attention to the need for more culturally sensitive and inclusive technological solutions.

## Data Availability

The raw data supporting the conclusions of this article will be made available by the authors, without undue reservation.

## Footnotes

^1^A cultural trauma occurs as the taken-for-granted foundations of individual and collective identity are shattered, setting in motion a discursive process to understand what happened, assign blame, and find pathways to repair an interpreted situation (Demertzis and Eyerman, 2020).
